# Seeing through Musculoskeletal Tissues: Improving In Situ Imaging of Bone and the Lacunar Canalicular System through Optical Clearing

**DOI:** 10.1371/journal.pone.0150268

**Published:** 2016-03-01

**Authors:** Ian M. Berke, Joseph P. Miola, Michael A. David, Melanie K. Smith, Christopher Price

**Affiliations:** Department of Biomedical Engineering, University of Delaware, Newark, Delaware, United States of America; National University of Ireland Galway, IRELAND

## Abstract

In situ, cells of the musculoskeletal system reside within complex and often interconnected 3-D environments. Key to better understanding how 3-D tissue and cellular environments regulate musculoskeletal physiology, homeostasis, and health is the use of robust methodologies for directly visualizing cell-cell and cell-matrix architecture in situ. However, the use of standard optical imaging techniques is often of limited utility in deep imaging of intact musculoskeletal tissues due to the highly scattering nature of biological tissues. Drawing inspiration from recent developments in the deep-tissue imaging field, we describe the application of immersion based optical clearing techniques, which utilize the principle of refractive index (RI) matching between the clearing/mounting media and tissue under observation, to improve the deep, in situ imaging of musculoskeletal tissues. To date, few optical clearing techniques have been applied specifically to musculoskeletal tissues, and a systematic comparison of the clearing ability of optical clearing agents in musculoskeletal tissues has yet to be fully demonstrated. In this study we tested the ability of eight different aqueous and non-aqueous clearing agents, with RIs ranging from 1.45 to 1.56, to optically clear murine knee joints and cortical bone. We demonstrated and quantified the ability of these optical clearing agents to clear musculoskeletal tissues and improve both macro- and micro-scale imaging of musculoskeletal tissue across several imaging modalities (stereomicroscopy, spectroscopy, and one-, and two-photon confocal microscopy) and investigational techniques (dynamic bone labeling and en bloc tissue staining). Based upon these findings we believe that optical clearing, in combination with advanced imaging techniques, has the potential to complement classical musculoskeletal analysis techniques; opening the door for improved in situ investigation and quantification of musculoskeletal tissues.

## Introduction

Cells of the musculoskeletal system (e.g. osteocytes, osteoblasts, osteoclasts, chondrocytes, and tenocytes; to name a few) reside within complex and often interconnected three-dimensional (3-D) environments [1–4]. The 3-D structure of these musculoskeletal cells, as well as their spatial interactions with the extracellular matrix and neighboring cells, are critical to their function [2,5–9]. Therefore, it should be well recognized that the removal of these cells from their native, in situ environment may alter cellular processes that are dependent on, or determined by cell and tissue architecture, cell-cell and cell-matrix interactions, and mechanosensory or mechanotransduction processes. In such cases, the environmental context that once was a critical signal in the regulation of cellular physiology is either physically removed upon their extraction from intact tissues or intrinsically missing from traditional two-dimensional (2-D) in vitro culture [10,11]. We believe that bioimaging approaches that directly interrogate the physiology of musculoskeletal cells and tissues in situ can provide structural and environmental context allowing for deeper insights into the cellular and molecular processes that regulate musculoskeletal health and disease. For example, through a series of in situ bioimaging and biomechanics studies we have previously quantified diffusive and load-induced molecular transport within the extra- and peri-cellular environments of several healthy and diseased murine musculoskeletal tissues [12–16] and thus provided better understanding of the mechanosensory environment within intact musculoskeletal tissues. While these studies relied heavily on techniques involving real-time empirical quantification of molecular transport processes in situ, the full realization of their findings required a fundamental understanding of in situ tissue and cellular architecture to interpret and model these processes [12,15,17–19]. Moving forward, our goal, and a goal of the musculoskeletal mechanobiology field in general, is to better quantify how complex in situ 3-D tissue and cell structure, and their interconnectedness, regulates the cellular physiology, homeostasis, and health of the musculoskeletal system.

Studying the role of cell architecture, cell-cell/cell-matrix interactions, and cellular processes in musculoskeletal physiology requires robust methodologies for the high resolution visualization of these properties in situ [20,21]. Presently, protocols for visualizing 3-D cellular structure in musculoskeletal tissues are costly, laborious, often result in relatively poor quality, and are typically performed in “non-intact” tissues. Several optical microscopy techniques described as “gold-standards” exist: i) reconstruction of serial sections (including paraffin, plastic, and cryo-sections), and ii) en bloc imaging (including plastic-embedded thick sectioning and serial milling). Reconstruction of serial sections requires thin sectioning of embedded or cryopreserved specimens, followed by assay-specific staining processes, microscopic imaging, and 3-D digital reconstruction [22–24]. These processes are time consuming, costly, and fraught with artifacts, such as registration issues, morphological distortion and sample loss [25]. As an alternative, en bloc imaging of plastic embedded musculoskeletal specimens preserves their 3-D cellular and tissue morphology, however, it still requires laborious processes of staining, processing, embedding, cutting, polishing, mounting, and confocal imaging [19,26]. While en bloc staining and imaging has benefits over serial section reconstruction, limitations still exist. These include: i) often-significant loss of specimens through destructive/subtractive techniques of specimen preparation, ii) inability to use certain fluorescent probes due to harsh embedding conditions [27], and importantly, iii) loss of image quality with increasing depth, often beyond as little as 40-um (personal observation), as a result of light scattering through specimen interfaces where tissue and embedding medium refractive indices (RI) are mismatched [28]. The development of serial milling based imaging techniques [29,30] has mitigated some of these issues, permitting submicron imaging of musculoskeletal tissues; however, milling techniques are time consuming and remain destructive to specimens. While these techniques for 3-D visualization and quantification have been applied with reasonable success in histomorphometric and cytoarchitectural studies of musculoskeletal tissues, they presently remain limited in their investigation flexibility and applicability to high-throughput in situ optical imaging.

Recently, advances in deep-tissue microscopy [31–33] suggest that a simple modification to en bloc imaging techniques could alleviate some limitations to in situ musculoskeletal optical imaging; namely, the optical clearing and mounting of tissues. Within the visible and near infrared spectrum tissues are generally low absorbing, highly scattering materials. Thus, light scatter typically represents the greatest limiting factor to successful deep-imaging in biological specimens [28,34]. In tissues, light scatter occurs wherever mismatches are found in the refractive indices (RI) of the materials through which light is passing (Table 1) [35], leading to a general loss of signal, a reduced depth of imaging penetration, and a decrease in image quality [28] across all modes of optical microscopy (e.g. transmitted light-, reflected light-, epifluorescence-, confocal-, and multiphoton-microscopy). Typically, these mismatches occur at the objective-to-air/immersion-, air/immersion-to-coverslip-, coverslip-to-mountant-, mountant-to-specimen-, and intraspecimen-interfaces. Of these, the mountant-to-specimen and intra-specimen-interfaces (including those between extracellular matrix, extracellular fluid, and intracellular fluid; Table 1) present the greatest challenges to successful imaging since they are of complex geometry and variable RI mismatches, and are typically not correctable using compensating optics. However, recent theoretical [36,37] and experimental [31–33,38–45] studies have demonstrated that by introducing optical clearing agents into the tissues one can decrease intra-tissue RI mismatches, decrease light scattering, and render biological specimens optically clear. As a result, optical clearing can drastically improve high-resolution, high-quality, in situ deep-tissue imaging [31–33].

**Table 1:** Refractive index properties of materials encountered during typical imaging of musculoskeletal tissue specimens.

Imaging Materials	RI	References
Water	1.33	Refractive Index Database—http://refractiveindex.com
Acrylic	1.49	″
Imaging Oil	1.52	″
Borosilicate Glass	1.54	″
**Biological Tissues**		
Skin	1.36	[46]
Cartilage	1.38	[47]
Muscle	1.39	[46]
Adipose	1.46	[46]
Bone	1.55	[48]
**Tissue Components**		
Interstitial Fluid	1.35	[49]
Intracellular Fluid	1.37	[49]
Cellular Organelles	1.38–1.41	[50,51]
Connective Tissue/ECM	1.47	[52]
Calcium Hydroxyapatite	1.63–1.65	[53]

To date, a large number of immersion-based optical clearing agents, with RIs ranging from 1.33 to 1.56, have been developed and tested in a variety of tissues, most notably neuronal/brain tissue, embryos, and skin [31,32,38–43,54]. Unfortunately, few of these techniques have been applied directly to the study of adult musculoskeletal tissues [55–57]. Only recently, has the water soluble clearing and radiographic contrast agent, Iohexol, been shown to improve imaging of the articular cartilage-subchondral bone interface using optical coherence tomography [57]; however, this study was restricted to imaging of gross macroscopic subchondral bone architecture. Additionally, Calve and Neu recently confirmed the ability of the fructose-based clearing technique, SeeDB, to improve confocal imaging of cellular features in isolated bovine osteochondral, meniscal, and ligamentous tissues [55,56], as well as select murine musculoskeletal tissues [55]. However, a systematic, side-by-side comparison of the ability of modern optical clearing techniques to improve deep-tissue imaging in intact musculoskeletal tissues, especially with regard to the architectural/morphological study of bone, has yet to be fully demonstrated.

Given the dependence of light scatter, and optical clearing within tissues, on the difference in the RI between the tissue and the optical clearing agent, we wished to test the hypothesis that use of optical clearing agents with higher RIs (that more closely match the RI of musculoskeletal tissues; up to 1.55) will significantly improve the optical clarity, confocal imaging depth, and 3-D imaging quality of musculoskeletal specimens. Thus, the goal of this study was to demonstrate and directly compare the in situ optical clearing properties of several recently developed optical clearing techniques (with RIs ranging from 1.45 to 1.56 Table 2) within intact murine musculoskeletal tissues, with a special emphasis on bone. In the present paper, we established the optical clearing properties of eight non-aqueous and aqueous optical clearing agents within murine musculoskeletal tissues, and identified several clearing agents that significantly improved optical imaging capabilities across a variety of investigational applications and imaging modalities when compared to “gold-standard” analysis techniques. Based upon these results we also provide recommendations for the use of optical clearing techniques within murine musculoskeletal tissues (especially intact bone), as well as illustrate the potential that optical clearing techniques hold for transforming the in situ study of tissue and cellular architecture, and cell physiology in musculoskeletal tissues.

**Table 2:** Properties of the optical clearing agents and clearing procedures utilized within the present study.

					Processing + Clearing Time^3^			
Clearing (and Mounting) Agents	Concentration^1^ and Molecular Weight (g/mol) of Final Clearing Agent	RI^2^	Aqueous vs. Non-Aqueous	Single- or Multi-step Procedure	Murine Bone Segments	Intact Murine Knee Joints	Cost of Clearing Agents^4^ ($US/mL)	References
Water^5^	100% Water (MW 18.02)	1.34	Aq	Single	Not Applicable	Not Applicable	Not Applicable	
Visikol	100% Visikol (Makeup and MWs are Proprietary)	1.45	Aq	Single	~24-hrs	4-d	$1.20	www.visikol.com
Clear T2	50% formamide (MW 45.04) + 20% PEG (MW ~8000)	1.45	Aq	Multi	~48-hrs	6-d	$0.41	[39]
Focus Clear	100% Focus Clear (Makeup and MWs are Proprietary)	1.46	Aq	Single	2-d	6-d	$78.00	www.cedarlanelabs.com
2, 2'-thiodiethanol (TDE)	97% TDE (MW 122.19) in water	1.47	Aq	Multi	2-d	4-d	$1.04	[41]
See Deep Brain (SeeDB)	80.2% D-fructose wt/wt (MW 180.16) in water	1.50	Aq	Multi	4-d	14-d	$0.29	[40]
Methyl salicylate (MS)	100% MS (MW 152.15)	1.51	Non-Aq	Multi	4-d	7-d	$0.31	[58]
Benzyl benzoate-benzyl alcohol (BABB)	25% benzyl alcohol (MW 108.14) + 75% benzyl benzoate (MW 212.24)	1.53	Non-Aq	Multi	4-d	7-d	$0.30	[43]
Tetrahydrofuran-dibenzyl ether (THF-DBE)	100% DBE (MW 198.26)	1.56	Non-Aq	Multi	4-d	7-d	$0.16	[42]
Acrylic^6^	85% methyl methacrylate (MW 100.12) + 15% n-butyl phthalate (MW 278.34) + 2% benzoyl peroxide (wt/vol) (MW 242.23)	1.49	Non-Aq	Multi	9-d	14-d	$1.13	[26]

^1^All concentrations are indicated as vol/vol unless otherwise noted

^2^RI values obtained from literature

^3^Values indicate approximate total time required for full processing and clearing protocol

^4^Reference provided is for the cost of clearing reagents only at the time of the study; cost of common processing reagents (e.g. ethanol, etc.) was not included

^5^Water refers to non-cleared samples immersed in deionized water

^6^Acrylic refers to the plastic embedding procedure; samples are infiltrated with the indicated agents and then polymerized within the solid support matrix

## Materials and Methods

### Animals

For the present study BALB/C mice (male and female) were bred and raised in house, and sacrificed between 16 and 20-weeks of age (n = 40). A subset of mice (n = 5), sacrificed at 8-weeks of age, were administered dynamic bone labels, calcein green (10-mg/kg) and alizarin red complexone (10-mg/kg), subcutaneously, 13- and 3-days prior to sacrifice, respectively. Mice were housed in standard laboratory cages (~7.75-inches wide x 12-inches deep x 6.5-inches tall) with a maximum of 5-mice per cage, separated by sex. The housing environment was enriched with clean nesting materials (Kraft paper nesting). Mice were allowed access ad libitum to a standard mouse chow (Prolab RMH 3000) and sterilized water, and were maintained on a 12-hour light-dark cycle in a climate controlled vivarium. At the indicated experimental time points mice were euthanized using carbon dioxide (CO_2_) asphyxiation followed by cervical dislocation and confirmation of respiratory and cardiac arrest.

#### Ethics statement

All animal handling and experiments and handling were carried out in strict accordance with the recommendations in the Guide for the Care and Use of Laboratory Animals of the National Institutes of Health. Animal use protocols were approved by University of Delaware’s Institutional Animal Use and Care Committee (Permit Number 1252).

#### Specimen preparation

Depending on the imaging procedure/technique arms that the musculoskeletal tissues were destined for, one of three types of tissue specimens were harvested/prepared. From the 16 to 20-week-old mice 1) intact right tibiofemoral (knee) joints (n = 18 joints), including the entire tibiae and the distal 4–5 mm of the femora were collected upon sacrifice for gross observation of joint and tissue clearing. The remaining portions of the right femoral diaphyses and whole left femora (~15.1-mm in length) from these and additional mice were 2) cut into ~2.5-mm thick, transverse segments using a low speed saw (Isomet, Buehler, IL) and diamond coated wafering blade (Buehler). From these segments, two to three samples, covering ~9–12 mm of the mid-diaphyseal femoral cortex were collected from each specimen (n = ~100 segments total). To reduce variability due to differences in bone size and shape, the central mid-diaphyseal segments, immediately below the greater trochanter, were reserved for unstained analysis of bone clearing and light transmission, while the segments proximal and distal to this were randomly assigned to the different staining and clearing arms of the study. From 8-week-old dynamic bone labeled mice 3) tibiofemoral complexes were disarticulated and intact femora and tibiae were collected and assigned to select staining and clearing arms of the study. Following isolation, all samples were fixed for 48-hours at 4°Celsius in neutral buffered formalin (z-Fix, Anatech Ltd, Battle Creek MI).

### Staining, processing, & clearing

After fixation, samples were washed in PBS and then assigned to their respective en bloc staining and/or optical clearing groups. Techniques utilized, and described briefly herein, were based upon modifications to well established protocols for tissue staining, processing, and clearing/mounting, all procedures were performed at room temperature, unless noted otherwise. Detailed experimental protocols including processing steps, conditions, and times (dependent on tissue specimen size) for staining and clearing are provided in the S1 Methods.

#### En bloc staining

Samples in the en bloc staining arm of the study (femoral bone segments) were stained in the ethanol soluble dye, basic fuschin [26], or with Osteochrome (Villaneuva’s Osteochrome Bone Stain; Polysciences, Inc., Warrington, PA) in order to visualize the architecture of the bone osteocyte lacunar-canalicular system. Following *en bloc* staining, both sets of specimens were then directed down selected clearing pathways. Specimens in the unstained arms of the study (including the dynamic labeling arm) were immediately directed down their requisite aqueous and non-aqueous clearing pathways following PBS washing.

#### Optical clearing

Five aqueous-compatible clearing techniques were investigated (Table 1): Visikol, ClearT2 [39], FocusClear, See Deep Brain (SeeDB) [40], and 2,2’-thiodiethanol (TDE) [38,41].

Visikol: Following washing in PBS, samples were directly transferred to and maintained in the proprietary clearing agent Visikol (Phytosys LLC, New Brunswick, NJ) until they achieved maximal clearance, and were then mounted in Visikol.

ClearT2: Clearing in ClearT2 involved immersion of samples in solutions of 25% formamide (FA) plus 10% polyethylene glycol (PEG) in PBS, then repeatedly in 50% FA + 20% PEG (ClearT2) till cleared, and then mounting in ClearT2.

FocusClear: Clearing in FocusClear (Cedarlane, Burlington, NC) was achieved by immersing the samples in FocusClear, a one-step proprietary clearing agent, till clear, followed by mounting in MountClear.

SeeDB: Clearing in SeeDB involved immersion in increasing concentrations of fructose in water (20–100% wt/vol) at room temperature, followed by immersion and mounting in 80.2% wt/wt fructose in water (SeeDB) at 50°Celsius.

TDE: Clearing in TDE involved the sequential immersion of the samples in increasing concentrations of 2,2’-thiodiethanol in water (10%, 25%, 50%, and 97% vol/vol). The final concentration of TDE can be varied to tune its refractive index; 97% TDE corresponds to a RI of 1.47; thus, specimens were immersed in 97% TDE until clear and then mounted in 97% TDE for imaging.

Three non-aqueous clearing techniques were investigated in this study (Table 1): methyl salicylate (MS) [59], benzyl alcohol-benzyl benzoate (BABB) [43,60], and tetrahydrofuran-dibenzyl ether (THF-DBE) [42]. For each of the non-aqueous techniques the samples underwent dehydration prior to clearing.

MS: Following dehydration in graded ethanols, the samples were immersed sequentially in 2-propanol, MS (a.k.a. Murray’s clear) till clear, and then mounted in MS.

BABB: Following dehydration in graded ethanols, samples were immersed in hexane, and then benzyl alcohol-benzyl benzoate (BABB; 1:3 vol/vol) till the samples were sank and were clear, followed by mounting in BABB.

THF-DBE: During THF-DBE processing samples were dehydrated in increasing concentrations of THF in water (50–100% vol/vol), followed by immersion in DBE until clear and were then mounted in DBE.

Additionally, a subset of samples underwent non-aqueous processing for plastic embedding in methyl methacrylate (acrylic) [26]. See S1 Methods for specifics of the plastic embedding procedure.

### Imaging

#### Visible light stereomicroscopy

Following clearing, unstained intact joints (n = 2 joints per clearing technique) and the unstained bone segments (n = 3 segments per clearing technique) were mounted in their final clearing/mounting agents and imaged via visible light stereomicroscope (Stemi2000c, Carl Zeiss, Inc.) to qualitatively assess the effect of clearing agents on the optical clarity of musculoskeletal tissues. Specimens were imaged in custom imaging baths placed atop a printed grid (spacing = 1.0 mm). Samples were imaged under illumination from both the front (LED ring-light, Carl Zeiss, Inc.) and behind (light pad, Logan Electric). Images were captured via a 24-megapixel digital camera (D3200, Nikon Inc.) attached to a PC using the DigiCamControl acquisition software (http://digicamcontrol.com/). Prior to imaging, bone segments were manually cleansed to remove all adherent tissue and to flush all marrow, leaving only the bone cortex for imaging. Intact joint specimens retained the majority of muscle and all connective tissue surrounding the joint. The optical clarity of various tissues within each specimen, including muscle, joint capsule, tendon/ligament, patella, meniscus, articular cartilage, calcified cartilage, cortical bone, and bone marrow, were qualitatively assessed. Tissues were described as opaque (not able to be seen through; not transparent), translucent (allowing light, but not detailed “images”, to pass through; semitransparent), transparent (allowing light to pass through so that objects behind can be distinctly seen; clear), or not determinable (obscured by overlying opaque or translucent tissues).

#### Light transmittance

In a subset of cleansed, 2.5-mm tall mid-diaphyseal femoral bone segments (n = 3–4 segments per clearing technique) the effect of optical clearing on light transmittance through bone was quantified using a light spectrophotometer (Cary 60 UV-Vis-NIR, Agilent Tech.). A paired assay was utilized to measure light transmittance (T), from the ultraviolet to near infrared spectrum (360–1090nm light), through the cortex prior to and after optical clearing. Use of the paired assay allowed for us to directly account for the presence of natural (albeit small) variances in femoral bone size and shape among our specimens. All specimens were imaged in a custom spectrophotometer cuvette/holder that positioned the bone segments so the spectrophotometer beam consistently and repeatedly passed through the samples in the anterior-to-posterior direction. For each clearing agent, transmittance readings were normalized to that of the clearing agent relative to pure water. For each bone specimen an average fold-increase in light transmittance between the cleared and uncleared state was calculated by dividing the average cleared transmittance (between 360 and 1090nm) by the respective uncleared transmittance.

#### Calculation of scatter coefficient

The Beer-Lambert Law describes the radiant intensity of light transmitted through a turbid material (i.e. tissue). The generalized form of the the Beer-Lambert Law, for both absorption and scatter at a given wavelength (*λ*) is:

$$I\left(\lambda \right)\;=\;I_{0}\left(\lambda \right)e^{-[\mu_{a}+\mu_{s}]z}$$ (1)

where *I* is the radiant power of monochromatic spectrophotometry beam leaving the sample, *I*_*0*_ is the reference power of monochromatic spectrophotometry beam, *μ*_*a*_ is the absorption coefficient, *μ*_*s*_ is the scatter coefficient, and z is the optical path thickness through the sample. Across the visible and near infrared spectrum, tissues typically exhibit low absorption, but high scatter; thus *μ*_*a*_ ≪ *μ*_*s*_, and, the Beer-Lambert Law can be approximated by:

$$\frac{I\left(\lambda \right)}{I_{0}\left(\lambda \right)}\;=\;e^{-\mu_{s}\cdot z}\;=\;T\left(\lambda \right)$$ (2)

where *I*(λ)/*I*_*0*_(λ) is defined as the transmittance (*T*) at the wavelength *λ*. Thus, the scatter coefficient (*μ*_*s*_), or approximate number of scattering events per cm, that occurred within uncleared and cleared bone segments could be calculated based upon the knowledge of measured transmittance through the sample and the path length of the scattering material. In the case of our murine bone samples the scattering material consisted of semi-cylindrical bone segments. Thus, the path length (*z*) of the scattering material was approximated by the sum of the anterior and posterior cortical thicknesses of each individual segment, Ct.Th_Anterior_ and Ct.Th_Posterior_, respectively, as measured by stereomicroscopic observation of each segment.

#### Confocal microscopy

En bloc stained and cleared bone specimens were subjected to 3D confocal imaging to assess imaging penetration depth and 3D micro-structural and imaging quality (LSM780 NLO, Carl Zeiss) and morphological changes (LSM880, Carl Zeiss). For all specimens, a 20x long working distance (WD = 1.70-mm), cover glass corrected, high numerical aperture (NA = 1.0) water immersion lens (W Plan-Apochromat 20x, N.A. 1.0, Carl Zeiss) or a 40x high numerical aperture, oil lens (Plan-Apochromat 40x, N.A. 1.4, WD = 0.13-mm, Carl Zeiss) was used for imaging. Samples were imaged in custom built imaging chambers covered with a #1.5 glass coverslip that allowed them to be imaged on either an upright LSM780 microscope or an inverted LSM880 microscope while simultaneously immersed in their respective clearing/mounting solutions. Zen image acquisition software (Carl Zeiss) was used to capture confocal images and z-stacks at 20x (0.414-um/pxl) to 120x (0.069-um/pxl) zoom. Dye/fluorophore appropriate fluorescence excitation and emission filtration settings were used for both 1-photon (1-P) and 2-photon (2-P) imaging. Scanning parameters were optimized to achieve the highest quality 3-D image stacks in the shortest scanning time. Detailed imaging parameters are described within the S1 Methods. Image analysis and 3-D reconstruction was performed using the Zen and Fiji [61] software analysis platforms.

### Data analysis

Data are presented as mean ± standard deviation. Statistical analyzes were performed using GraphPad Prism version 5.0 for Mac (GraphPad Software, San Diego California USA). Specific statistical tests and parameters utilized are described fully in the results section.

## Results

### Improved visible-light imaging of musculoskeletal tissues following optical clearing

**Fig 1:**
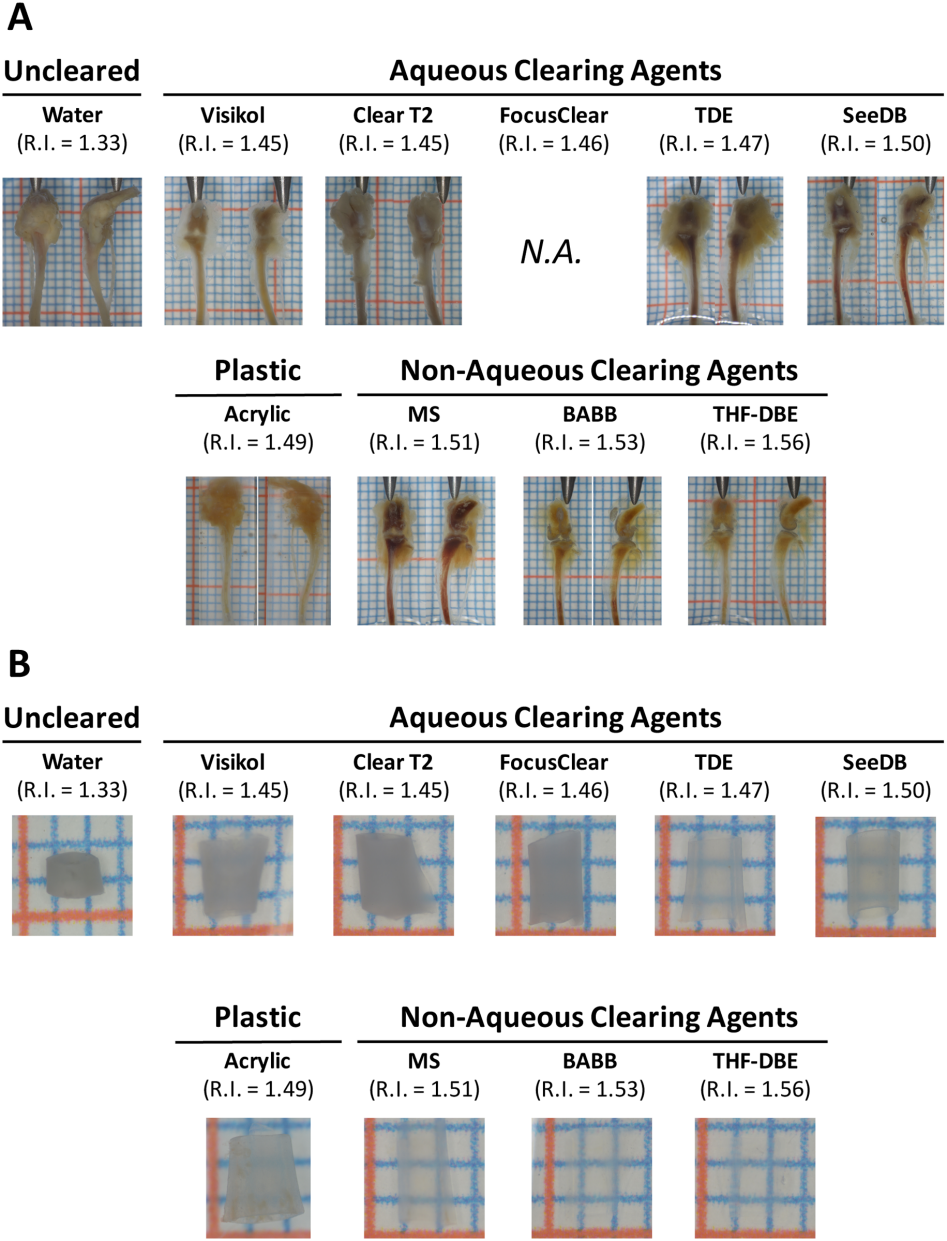
Stereomicroscopic appearance of musculoskeletal tissues following optical clearing in either aqueous or non-aqueous clearing agents, or embedding in plastic. A) Visual appearance of intact tibiofemoral joints following optical clearing. Non-aqueous clearing agents (MS, BABB, THF-DBE; RI > 1.51) resulted in the most appreciable clearing of musculoskeletal tissues, including muscle, connective tissue, cartilage, and bone. Scale bar indicates 2-mm; joints are shown in the anterior (left images) and lateral (right images) views. Focus Clear was not tested in intact knee joints. B) Visual inspection of cleansed, marrow flushed, and optically cleared mid-diaphyseal femoral bone segments. Clearing agents with RI > 1.47 demonstrated an appreciable ability to clear bone segments. Those agents with RI > 1.51 effectively rendered bone segments transparent. Scale bar indicates 1-mm.

The different clearing/mounting protocols tested in this study demonstrated variable ability to optically clear musculoskeletal tissues when viewed under 5x visible light stereomicroscopy. From a qualitative perspective, musculoskeletal tissues, including muscle, connective tissue, and bone from uncleared murine tibiofemoral (knee) joints immersed in water (RI = 1.33) appeared opaque under stereomicroscopic observation, with only exposed tissue surfaces being observable (Fig 1 & Table 3). The use of clearing agents with relatively low refractive indices (RI = 1.45–1.50) resulted in minor improvements to tissue clearing (mainly in the muscle and joint capsule) compared to uncleared controls (Fig 1A). However, it was grossly apparent that clearing solutions with higher refractive indices (RI = 1.51–1.54) were very effective in optically clearing musculoskeletal tissues (Fig 1A), especially with regard to muscle, joint connective tissue, and bone. Based upon this qualitative analysis it was apparent that non-aqueous, higher RI clearing techniques were better at clearing complex musculoskeletal organs, like intact knee joints, than their aqueous counterparts. Stereomicroscopic observation of the effect of the various clearing agents on the optical clarity of isolated and cleansed mid-diaphyseal femoral bone segments demonstrated similar qualitative findings (Fig 1B). Clearing agents with RIs of 1.46 or less resulted in nearly opaque bone, RIs of 1.47 to 1.51 resulted in increasingly translucent bone segments, and RIs of 1.51 to 1.56 produced increasingly transparent bones. For comparison, the qualitative clarity of intact knee joints immersed and embedded in plastic (the en bloc “gold standard” for current bone morphological/architectural analysis, RI = 1.49) was equivalent to that of the aqueous clearing agents, while the clarity of plastic embedded bone segments fell between the aqueous and non-aqueous clearing agents (Table 3).

**Table 3:** Qualitative visual description of tissue clarity following the optical clearing of intact knee joints.

			Qualitative Assessment of the Visual Clarity of Musculoskeletal Tissues Following Optical Clearing								
Clearing & Mounting Method		Mountant RI	Muscle	Joint Capsule	Tendon/ Ligament	Patella	Articular Cartilage	Meniscus	Calcified Cartilage	Cortical Bone*	Bone Marrow
**Un-cleared**	Water	1.33	Opaque	Opaque	Opaque	N.D.	N.D.	N.D.	N.D.	Opaque	N.D.
**Aqueous Methods**	Visikol	1.45	Translucent	Translucent	Translucent	Opaque	N.D.	N.D.	N.D.	Translucent	Opaque
	Clear T2	1.45	Opaque	Opaque	Opaque	N.D.	N.D.	N.D.	N.D.	Translucent	Opaque
	FocusClear	1.46	*Not evaluated (N*.*E*.*) due to high cost of clearing reagents (~$US78/mL)*							Translucent	*N*.*E*.
	TDE	1.47	Translucent	Translucent	Translucent	Opaque	Translucent	N.D.	N.D.	Translucent	Opaque
	SeeDB	1.5	Translucent	Translucent	Translucent	Translucent	Transparent	N.D.	Opaque	Translucent	Opaque
**Non-Aqueous Methods**	Acrylic	1.49	Opaque	Translucent	Translucent	N.D.	N.D.	N.D.	N.D.	Translucent	Translucent
	MS	1.51	Transparent	Transparent	Transparent	Translucent	Transparent	Translucent	Opaque	Transparent	Translucent
	BABB	1.53	Transparent	Transparent	Transparent	Transparent	Transparent	Transparent	Translucent	Transparent	Translucent
	THF-DBE	1.56	Transparent	Translucent	Transparent	Transparent	Transparent	Transparent	Translucent	Transparent	Translucent

Opaque = defined as "not able to be seen through; not transparent" (Oxford American College Dictionary)

Translucent = defined as "allowing light, but not detailed images, to pass through; semitransparent" (Oxford American College Dictionary)

Transparent = defined as "allowing light to pass through so that objects behind can be distinctly seen; clear" (Oxford American College Dictionary)

N.D. = Not determinable (obscured by overlying opaque or translucent tissues)

* = Confirmed in individual cleansed and marrow-flushed bone segments

The effect of optical clearing on gross changes in musculoskeletal tissue morphology was also assessed. From a qualitative perspective, gross changes in the morphology of musculoskeletal tissues from intact murine joints were only observed for BABB (shrinkage of muscle) and Visikol (swelling of muscle) following clearing; the remaining clearing agents exhibited no readily observable changes in the gross soft-tissue morphology of intact murine joints (not shown; clearing of intact joints by FocusClear was not tested). The effect of clearing on the morphology (size and shape) of non-decalcified, murine mid-diaphyseal femoral bone segments was assessed, qualitatively, in a pair-wise manner using digital calipers prior to and again following optical clearing. Optical clearing had no effect on the size (anterior-posterior and medial lateral Feret diameter; S1A & S1B Fig) or shape (AP width/ML width; S1C Fig) of non-decalcified bone segments (paired t-test, p<0.05; GraphPad Prism).

### Improved light transmission following optical clearing

**Fig 2:**
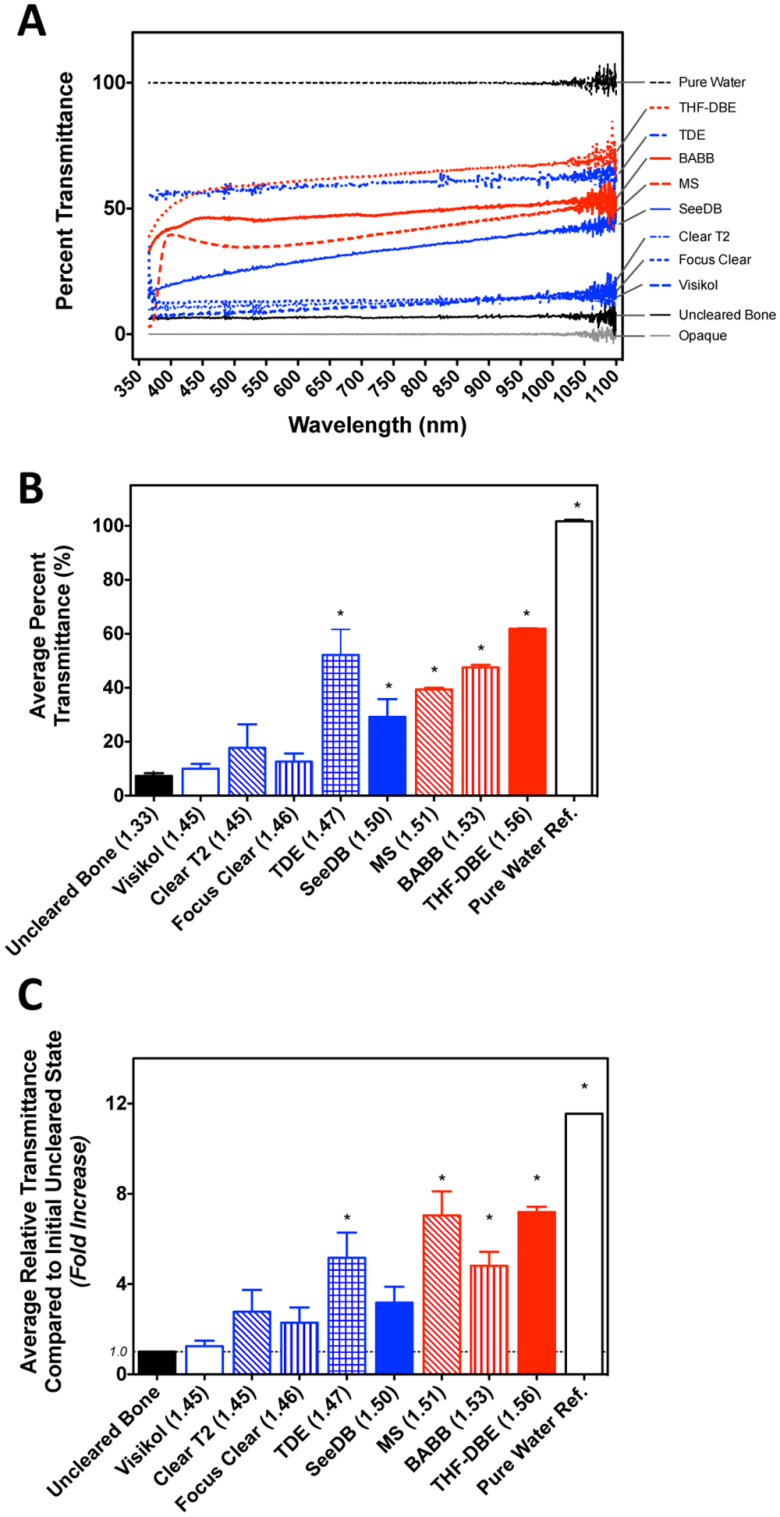
Changes in the visible light transmittance properties of intact, cleansed bone segments following optical clearing. A) Optical clearing agents increased the percentage of light (360–1090nm) that passed through cleansed mid-diaphyseal femoral bone segments compared to uncleared bone. For each clearing agent a representative individual transmittance spectral curve is shown. All data was normalized to that of the clearing agent relative to pure water. B.) Quantification of mean (broadband) light transmittance through bone segments following optical clearing. These data are derived from readings on three samples within each clearing agent group. Asterisks (*) indicate a significant difference from that of uncleared bone (one-way ANOVA, Tukey’s post hoc test, p<0.05; GraphPad Prism). C) Relative increase in light transmittance through murine bone segments following optical clearing. Asterisks (*) indicate a significant difference from no change in relative light transmittance (one sample t-test against a theoretical value of 1.0, p<0.05; GraphPad Prism). In each panel data are shown in order of increasing RI values, red denotes non-aqueous clearing procedures and blue indicates aqueous procedures.

The effects of the selected optical clearing procedures on the optical clarity of bone tissue was further confirmed by quantifying the transmittance of light through cleansed mid-diaphyseal femoral bone segments in a pairwise manner. To accommodate for slight variation in bone size and shape (average anterior-posterior width = 1.166 ± 0.106-mm, average medial-lateral width = 1.821 ± 0.163-mm; n = 24 segments; S1A & S1B Fig) light transmittance through the bone segments was quantified prior to and after optical clearing using a UV-Vis spectrophotometer. Prior to clearing, the mean bone segment transmittance, defined as the average percent light transmitted through the sample across the measured spectrum (λ = 360–1090nm), was 7.4±4.5% (n = 24 samples), indicating that uncleared bone segments were largely opaque (Fig 2). Following clearing, the mean percent transmittance of light through the intact bone segments increased for all of the agents tested (10–62%, Fig 2A & 2B). Pair-wise calculation of the relative increase in light transmittance following clearing (*T*_*Cleared*_*/T*_*Uncleared*_) confirmed the ability of higher-RI clearing agents (R.I. > 1.47) to significantly improve the transmittance of UV and visible light through cortical bone (Fig 2C and Table 4; one sample t-test against a theoretical value of 1.0 indicated no change in transmittance, p<0.05; GraphPad Prism).

**Table 4:** Quantification of the average fold increase in broadband light transmittance observed in cleansed, intact bone segments following optical clearing. Measurements were performed in a paired manner; transmittance was quantified prior to clearing, and again following clearing and used to calculate the increase in transmittance due to clearing.

	Aqueous Clearing Agents					Non-aqueous Clearing Agents		
	Visikol (n = 3)	ClearT2 (n = 3)	FocusClear (n = 3)	TDE (n = 3)	SeeDB (n = 3)	MS (n = 3)	BABB (n = 3)	THF-DBE (n = 3)
R.I.	1.44	1.45	1.46	1.47	1.5	1.51	1.53	1.56
Fold Increase in Transmittance Following Clearing (Averaged over 360–1090nm)	1.25±0.41	2.77±1.68	2.29±1.17	5.17±1.94*	3.17±1.23	7.04±1.85*	4.81±1.08*	7.19±0.42*

* = Significantly different from a theoretical value of 1.0 (i.e. no change in relative transmittance, one sample t-test, p<0.05; GraphPad Prism)

**Fig 3:**
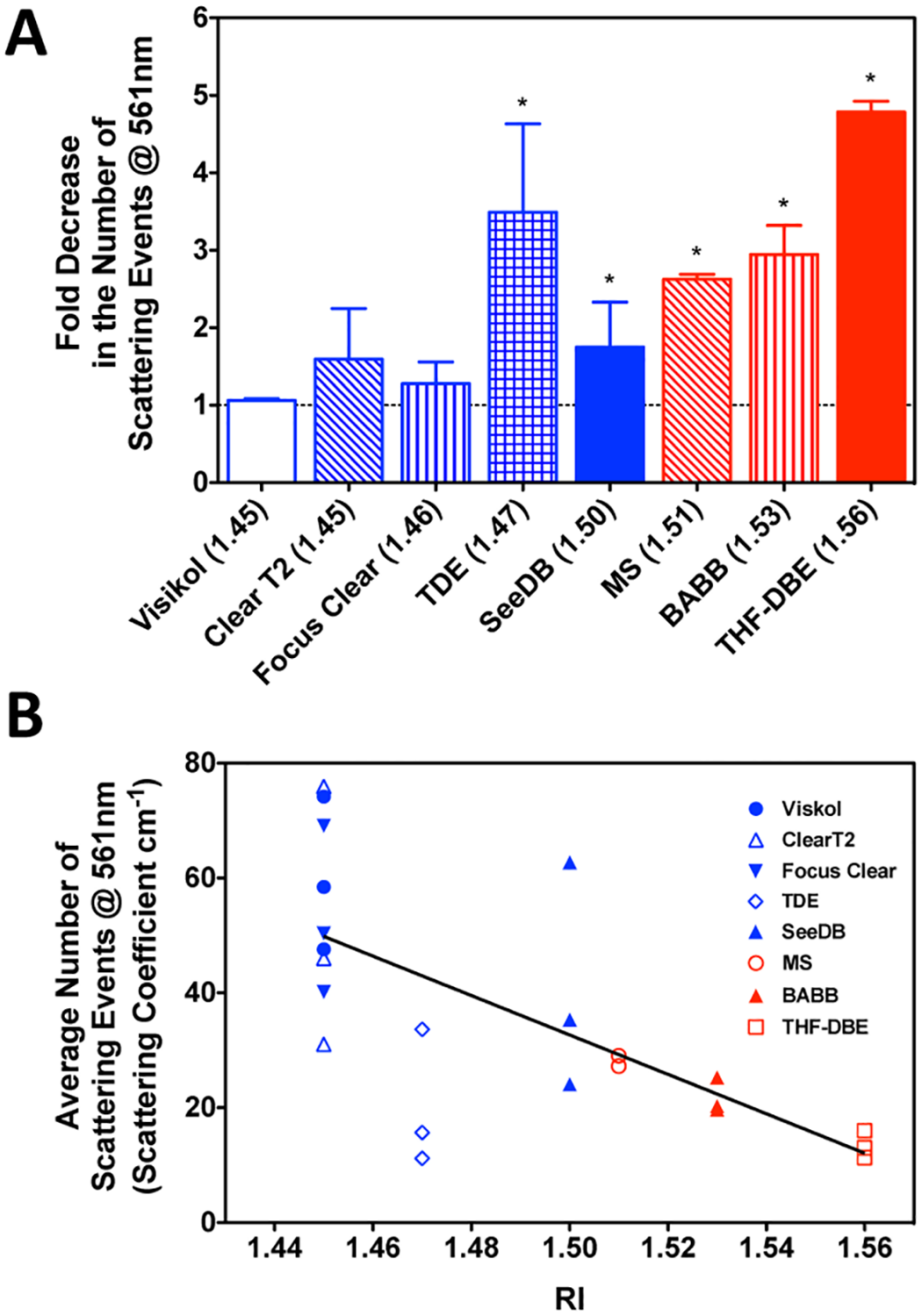
Characterization of light scattering within intact, cleansed bone segments following optical clearing. A) Fold decrease in the number of scattering events (at 561-nm) within murine bone segments following optical clearing. Asterisks (*) indicate a significant difference from no change in number of scattering events (one sample t-test against a theoretical value of 1.0, p<0.05; GraphPad Prism). Data are shown in order of increasing RI values, red denotes non-aqueous clearing procedures and blue indicates aqueous procedures. B) Relationship between the average number of scattering events predicted within cleared bone and the refractive index (RI) of the clearing/mounting agent. Linear regression y = -343.2*RI + 1.595, r^2^ = 0.457; GraphPad Prism).

Furthermore, extension of the Beer-Lambert law [*T(λ) = e^-μ*_*s*_*•z*] was utilized to estimate the change in the number of light scattering events (*μ*_*s*_; scatter coefficient [cm^-1^]) observed within our cylindrical murine bone segments following clearing. Quantification of scattering coefficients prior to and following optical clearing were performed for each specimen using pairwise light transmittance data at four commonly utilized UV and visible light wavelengths (405, 488, 561, 633-nm). The paired analysis procedure allowed for accommodation for minor variability in the optical path length among individual samples (path length (*z*) approximated by Ct.Th_Anterior_ + Ct.Th_Posterior_ = 0.401 ± 0.054-mm, as assayed by stereomicroscopy). Prior to clearing, the average scattering coefficient of murine bone across the selected UV to red visible light wavelengths was 66.10-cm^-1^, and did not exhibit significant wavelength dependence in the visible range (Table 5; one-way ANOVA, p<0.05; GraphPad Prism). Immersion in each of the optical clearing agents tested led to a subsequent decrease in the calculated scattering coefficient; with this reduction reaching significance for TDE, SeeDB, MS, and BABB (Fig 3A and Table 5; paired t-test, p<0.05; GraphPad Prism). Additionally, the observed decrease in scattering was largely inversely proportional to the refractive index of the clearing agent (Fig 3B).

**Table 5:** Quantification of the effect of optical clearing on the average number of light scattering events (scattering coefficient; cm^-1^) within murine bone segments.

		Un-cleared Samples	Aqueous Clearing Agents					Non-aqueous Clearing Agents		
	Wavelength	Water (n = 24)	Visikol (n = 3)	ClearT2 (n = 3)	FocusClear (n = 3)	TDE (n = 3)	SeeDB (n = 3)	MS (n = 3)	BABB (n = 3)	THF-DBE (n = 3)
R.I.		1.33	1.44	1.45	1.46	1.47	1.5	1.51	1.53	1.56
Number of Scattering Events (Scattering Coefficient μ_s_; cm^-1^)	405nm	66.31±4.32	64.23±16.03	52.62±22.80	55.48±15.95	22.51±13.39*	48.98±27.23	25.74±2.41*	20.45±2.57*	18.36±2.53*
	488nm	66.35±4.25	62.09±14.59	51.70±22.69	54.39±14.78	21.24±12.80*	44.09±22.41	28.14±1.28*	20.75±2.76*	14.45±2.51*
	561nm	66.31±4.12	60.07±13.38	51.00±22.85	53.23±14.68	20.18±11.89*	40.70±19.88*	28.15±1.28*	21.75±3.05*	13.45±2.38*
	633nm	65.45±3.87	57.07±11.72	50.01±22.86	50.88±13.15	19.51±11.23*	37.68±17.36*	27.28±1.67*	21.58±3.54*	12.69±2.31*

* = *Significantly different from number of scattering events in the un-cleared state of the samples* (paired t-test, p<0.05; GraphPad Prism)

### En bloc confocal 3-D dynamic histomorphometry

**Fig 4:**
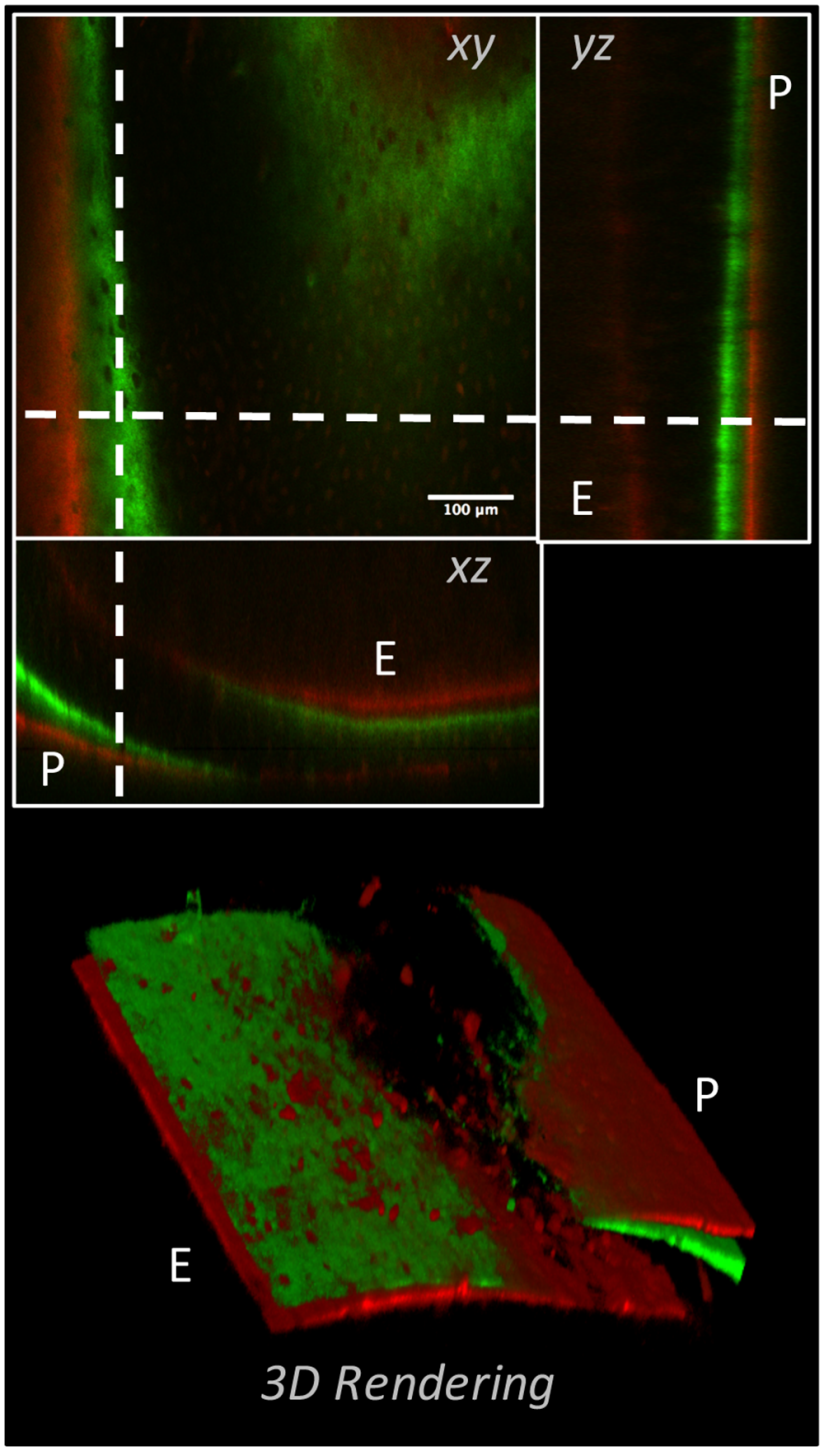
3-D representation of dynamically labeled cortical bone from an adolescent (8weeks of age) murine femur. The left panel shows three orthogonal views of a modeling/growing region of cortex in the femoral diaphysis of an adolescent mouse that was administered calcein green (green) and alizarin red complexone (red) 13- and 3-days prior to sacrifice, respectively. Both double and single labeled surfaces could be observed on the periosteal (P) and endosteal (E) surfaces. The right panel shows a volume rendered representation of the same data, illustrating the ability to visualize both double and single labeled appositional fronts, as well as discrete regions of bone formation “nodules” throughout the cortex.

Using a select set of non-aqueous (MS, BABB) and aqueous (Visikol, SeeDB) clearing techniques, we demonstrated the ability to visualize, via confocal microscopy, 3-D appositional growth in intact non-decalcified bone specimens from adolescent mice that were administered dynamic bone labels. The best results were obtained through the use of BABB, and are illustrated in Fig 3. Imaging of intact bone was restricted to the anterior-lateral cortex of the mid-diaphysis of the femur, as this region is known to exhibit high-levels of bone modeling during adolescent growth [62]. Using both 1- and 2-photon microscopy, we were able to clearly visualize the presence of bone formation via dynamic labeling (both double and single labels), within intact femoral bone cleared with BABB. Dynamic labels were readily observed as both continuous mineralization fronts and discrete bone formation nodules upon both the periosteal and endosteal surfaces of the femoral mid-diaphysis (Fig 4 and S2 Fig). In addition, labels were directly observed within the more complex trabecular bone of the third trochanter (S2 Fig). Animations of 3-D rendered dynamic bone labeling in BABB-cleared murine femora are provided in the Supplemental Movies (S1–S6 Movies)

### Improved 3-D osteocyte visualization in stained and cleared bone

**Fig 5:**
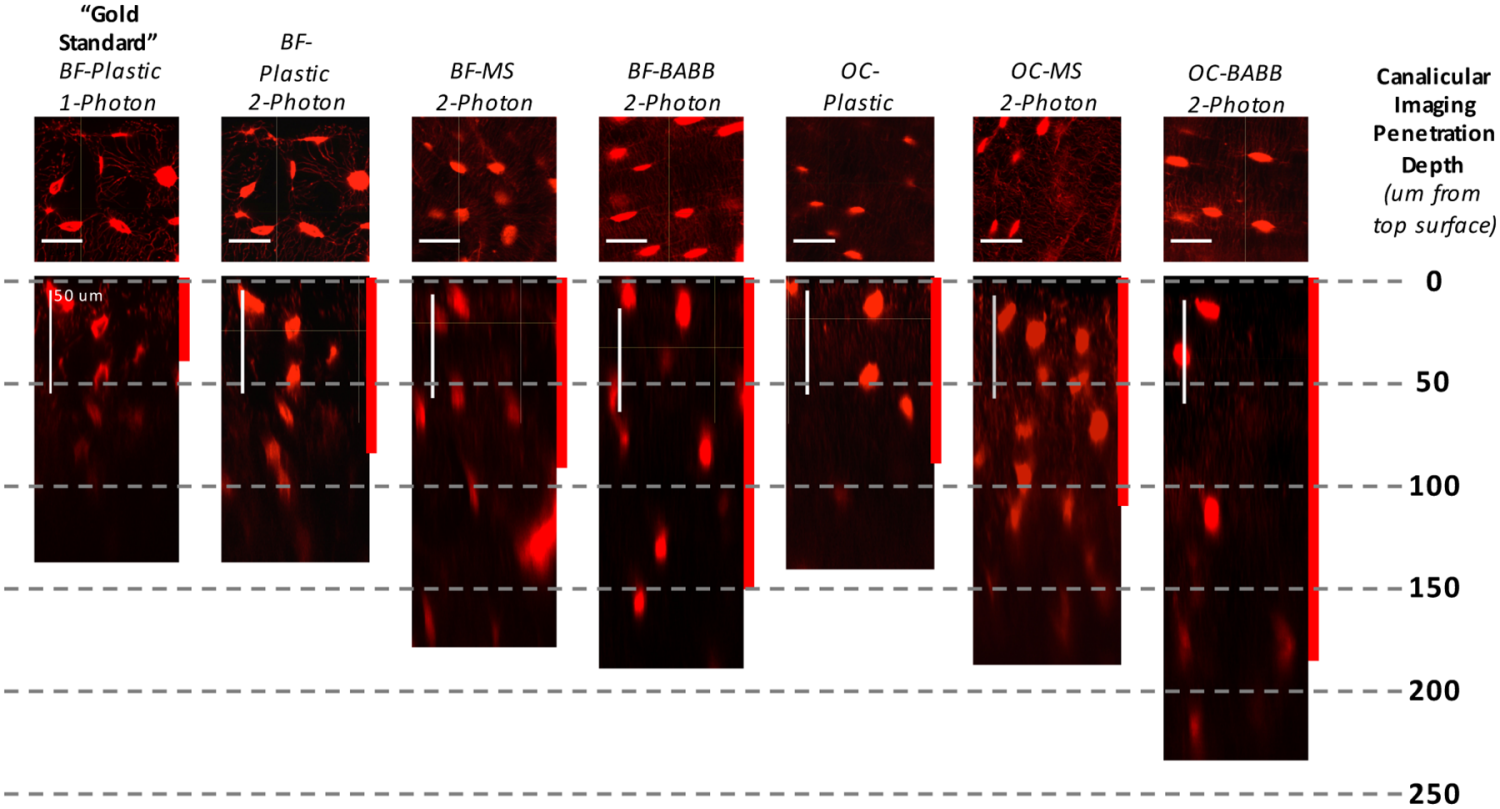
Visualization of bone’s 3-D lacunar canalicular system following non-aqueous optical clearing and mounting. Results from the historical “gold-standard” technique of basic fuschin (BF) stained plastic embedded one-photon confocal microscopy are shown in the leftmost panel. Approximately 45-um of imaging penetration was achieved before canalicular detail was qualitatively lost in plastic embedded BF samples (shown by the red bar indicating “canalicular imaging penetration depth”). Results obtained following subsequent refinements in the en bloc imaging techniques that included optical clearing using MS or BABB, incorporation of two-photon microscopy, and the use of Villaneuva’s Osteochrome Bone Stain instead of BF, are shown in the panels to the right. The greatest improvements were achieved though the use of Osteochrome, BABB, and 2-P imaging. Similarly, with the “gold-standard” technique, visualization of lacunar details was restricted to ~50-um in depth due to optical “shadowing” within lacuna (compare the two leftmost panels). Optical clearing and 2-photon imaging also drastically improved the imaging of lacunar detail at depth.

**Fig 6:**
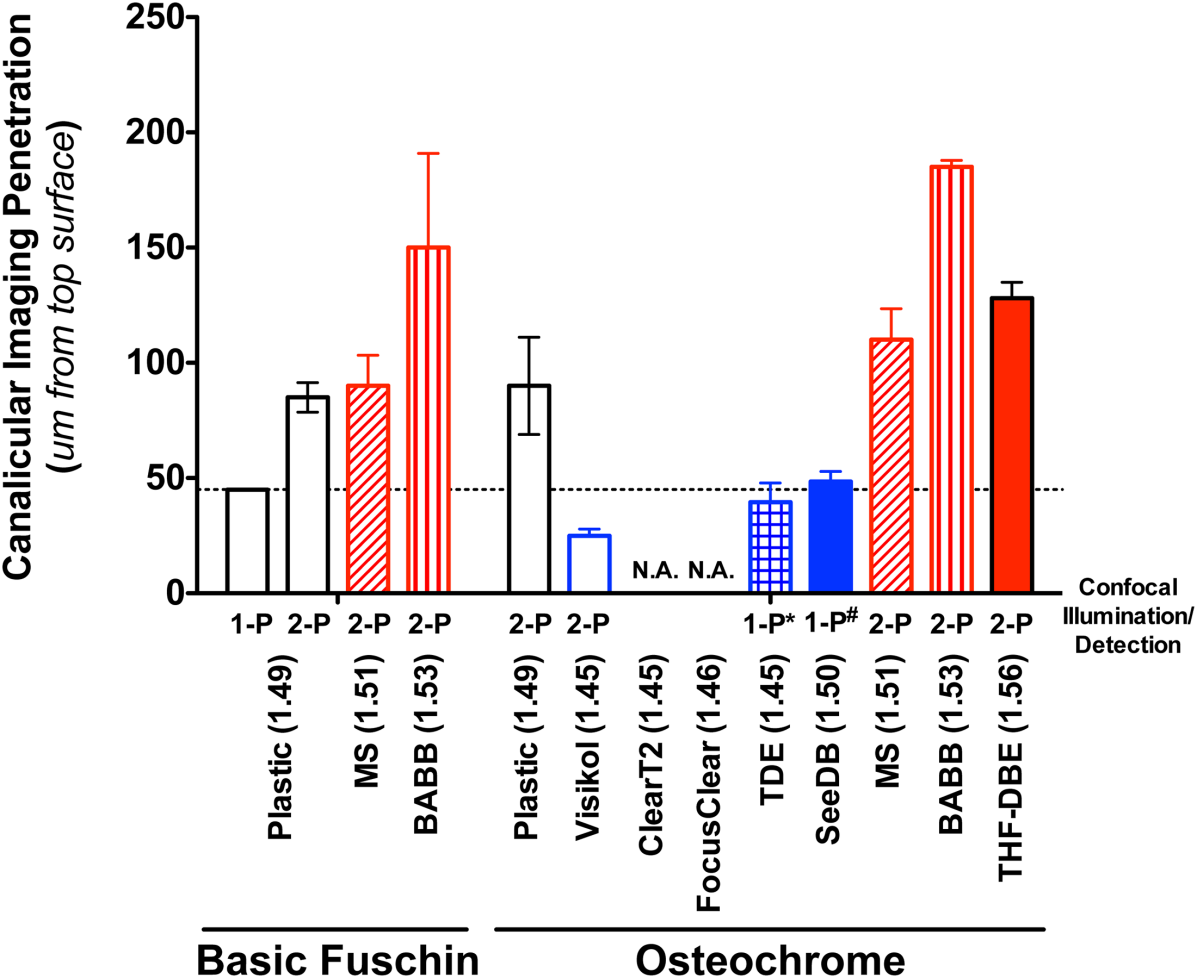
Quantification of bone’s canalicular confocal imaging penetration following en bloc staining and optical clearing. Non-aqueous clearing agents (MS, BABB, and THF-DBE) showed an appreciable ability to improve the visualization of fine canalicular detail (features on the order of 500 to 1000-nm in diameter) at depths 2 to 4-times that of the traditional “gold-standard” technique (plastic embedded 1-photon). Data represent the mean ± SD depth at which imaging of discrete canalicular structure is lost in stained, cleared, and imaged samples (n = 3–4 samples per clearing agent). 1-P indicates 1-photon imaging. 2-P indicates 2-photon imaging. ClearT2 and FocusClear were incompatible with Osteochrome staining, while TDE and SeeDB were incompatible with 2-photon imaging (*). Statistical analyses were not performed given the subjective nature of defining canalicular imaging penetration depth.

The application of select optical clearing agents to en bloc stained bones significantly increased our ability to visualize the osteocyte lacunar canalicular system (LCS) within intact bone specimens (Fig 5). Use of the “gold standard” technique of BF en bloc staining, plastic embedding, thick sectioning, and surface polishing followed by 1-photon 3-D confocal microscopy allowed for visualization of fine canalicular-level detail, (referred to as canalicular imaging penetration depth) only to a depth of ~45-microns (Fig 6). In addition, larger-scale lacunar-level detail (referred to as lacunar imaging penetration) also fell off rapidly at depths >50-microns due to optical shadowing under densely stained osteocyte lacuna/cell bodies (see cupping under lacuna in leftmost panel of Fig 5). Use of 2-photon confocal imaging improved canalicular and lacunar imaging penetration nearly 1.5-fold in BF stained plastic embedded samples (Figs 5 & 6); however, lacuna imaging at depth suffered similarly in the plastic embedded samples. The use of two much-simplified non-aqueous optical clearing techniques (MS and BABB) increased both canalicular and lacunar imaging penetration depth ~2–3 fold (Figs 5 & 6). Furthermore, a simple alteration to en bloc staining techniques, namely the use of Villaneuva’s Osteochrome Bone Stain in place of BF, followed by MS, BABB, or THF-DBE clearing procedures resulted in further improvements in the 2-photon visualization of fine, canalicular-level, and coarse, lacunar-level LCS details (ClearT2, and FocusClear were found to be incompatible with Osteochrome staining; TDE and SeeDB were compatible with 1-photon but not 2-photon imaging). Using Ostechrome in combination with MS or BABB clearing and mounting resulted in an improvement in canalicular imaging penetration depth of 2.4 to 4.1-fold, respectively (Figs 5 & 6). Lacunar imaging penetration in MS or BABB cleared samples often approached ~1-mm (not shown). Furthermore, the beneficial effects of optical clearing on the visualization of cyto-architecture within intact bone was exemplified in our ability to observe fine osteocyte and vascular morphology detail throughout the full thickness of intact, Osteochrome stained, BABB cleared murine calvarial and femoral cortices (~90 to 250um thick, respectively; S7 & S8 Movies).

**Fig 7:**
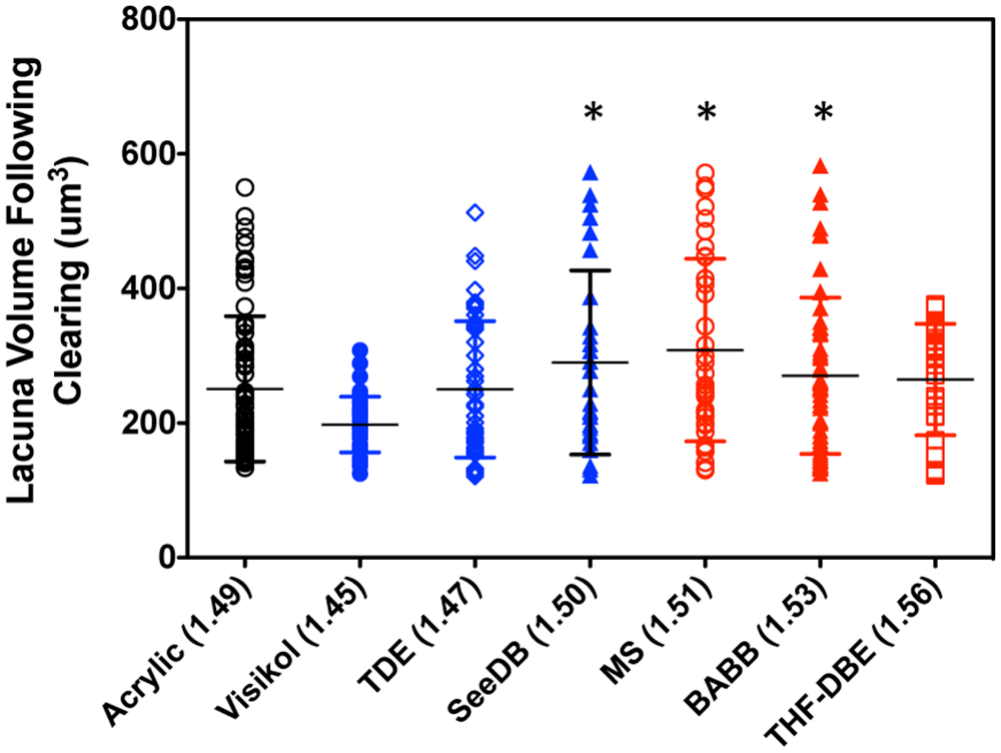
Quantification of effect of optical clearing on the volume of lacuna in Osteochrome stained bone segments. Following Osteochrome staining and optical clearing the volume of bone lacuna, imaged via 1-photon microscopy, was quantified and compared to that of stained and acrylic (plastic) embedded samples (“gold-standard”). No of the lacunar volumes measured for the tested clearing agents differed significantly from the acrylic embedded samples. Data are shown via a dot plot, horizontal bars indicate mean ± S.D. Astericks (*) indicates the specified clearing agent groups are significantly different from the Visikol group.

Lastly, the effect of the various optical clearing procedures on the architecture of the bone LCS was assessed. Using a high numerical aperture oil immersion objective (EC Plan-Apochromat 40x), a confocal microscope (Zeiss LSM 880) was used to capture high-resolution z-stacks of a 106.2-um by 106.2-um by 50.0-um tall volume of interest (0.104-um x 0.104-um x 0.532-um voxel size) of the LCS of Ostechrome stained and optically cleared mid-diaphyseal murine bone segments. Image capture was restricted to the posterior to medial quadrant of the cortex to control for possible spatial variances in osteocyte distribution and size. Average osteocyte lacuna size and distribution was calculated for each sample and clearing agent tested (n = 3–4 segments per agent, n = 28–72 lacuna per agent) on a volumetric basis from reconstructed and manually segmented confocal z-stacks in using the 3D Object Counter plugin within Fiji. While there were minor differences in the distribution of LCS lacunar volume among the clearing agents (Fig 7), average lacunar volume following optical clearing did not differ from that of the acrylic (i.e. plastic) embedded samples (One-way ANOVA followed by Bonferroni’s Post-hoc Test, p<0.05; GraphPad Prism). Between groups, only lacunar volumes in the Visikol cleared samples were found to differ significantly from any other group (smaller than the SeeDB, MS, and BABB cleared groups); this observation may be the result of a generally poorer clearing performance for Visikol in our bone samples. Lacunar volume in ClearT2 and FocusClear treated bone segments could not be assessed since Osteochrome LCS staining was not observable by confocal microscopy following ClearT2 or FocusClear clearing (not shown).

## Discussion

Cells of the musculoskeletal system reside within complex 3-D environments where their function and homeostasis depends critically on their interactions with neighboring cells and the surrounding ECM [1–4]. Studies directly interrogating the complex 3-D morphology, architecture, interactions among, and functions of musculoskeletal cells in situ have risen to the fore in the study of musculoskeletal physiology, homeostasis, and disease [12–16,63–70]. Numerous imaging techniques, including electron microscopy, serial sectioning and reconstruction, and en bloc imaging have been utilized to visualize and quantify 3-D musculoskeletal cell and tissue properties [3,17,19,71,72] in situ. Notwithstanding their successes, these techniques exhibit significant limitations regarding cost, ease of use, loss/destruction of samples during preparation, and limited ability for high-throughput, whole-tissue-level visualization.

Optical imaging, using modalities such as confocal microscopy, represents a flexible, and near ubiquitous research tool that could be leveraged to visualize the complex 3-D architecture and interactions among musculoskeletal tissues and cells in situ. Unfortunately, biological tissues are highly scattering, turbid materials [28] due to the presence of refractive index (RI) mismatches between interstitial fluid, intracellular fluid, cells/organelles, and the extracellular matrix (Table 1). This phenomena is especially prevalent in musculoskeletal tissue due to the large mismatches between the RI of interstitial/intracellular fluids (~1.35–1.37) [49] and the RIs of cartilage (~1.40) [47], connective tissues (~1.47) [52], and bone (~1.55) [48]; thus optical imaging of musculoskeletal tissues often suffers, and is limited by, scatter-dependent loss of signal at relatively shallow tissue depths (~40–50um) [49].

Recently, advances in deep-tissue microscopy [31–33] demonstrate that the simple immersion of turbid biological tissues in optical clearing agents can reduce light scatter in biological tissues through the mechanism of refractive index matching (by direct RI matching of the immersion agent and the tissue scatters [49] or by tissue dehydration [73,74]). Reduction of RI mismatches in tissues leads to a decrease in light scattering and an increase in light penetration within tissues; thus signal loss during imaging is reduced and morphological detail is better preserved [28]. To date, numerous clearing agents, with RIs ranging from that of water (1.33) to THF-DBE (1.56), have been developed [38]. Some of these clearing and mounting methods have been known to the microscopy community for decades [44], while others have only been recently developed and validated to meet the needs of modern bioimaging applications.

Historically, investigations utilizing optical clearing techniques have been directed toward studies of developmental, neural, and through-skin imaging [75]; the application of optical clearing techniques to the direct study of musculoskeletal tissue remains nascent. A limited number of studies have investigated the effect of individual optical clearing agents on musculoskeletal tissues. Several studies have looked into the potential of bulk clearing of musculoskeletal tissue (Iohexol [57], glycerol [76], skull optical clearing solution [SOCS] [77]) for visualizing underlying tissues and structures; however, the direct study of musculoskeletal tissue and cellular architecture was beyond the scope of these studies. Recently, two studies demonstrated the effect of the fructose-based (aqueous) clearing agent SeeDB on improvements in the in situ imaging of musculoskeletal cellular architecture (bovine cartilage, ligament and meniscus, and intact murine skulls, limbs, and bone) [55,56]. However, these studies were restricted to the investigation of only a single clearing agent. As a result, a comprehensive comparison of the effects of different optical clearing techniques on musculoskeletal tissue clearing and imaging has yet to be demonstrated.

The purpose of this study was to demonstrate a systemic, side-by-side comparison of the ability of eight aqueous (Visikol, Clear T2, TDE, SeeDB) and non-aqueous (MS, BABB, THF-DBE) optical clearing agents (Table 2) to render intact murine musculoskeletal tissues optically clear (transparent) and thereby improve their in situ, en bloc deep-tissue imaging performance. An emphasis was placed on the study of murine musculoskeletal tissue, and emphasizing bone, due to the ubiquitous use of mouse models in the investigation of disease-, injury-, and genetic-based causes (and treatments) of musculoskeletal disease. Altogether, we identified that select optical clearing agents had the ability to drastically improve imaging capabilities across a range of imaging applications, including visible light stereomicroscopy of unstained samples, transmitted light spectrophotometry of unstained samples, and confocal microscopy of dynamically labeled or en bloc stained bone samples.

Qualitative stereomicroscopic observation of cleared, intact tibiofemoral joints demonstrated that complex, multi-tissue musculoskeletal specimens were preferentially cleared by clearing agents having RIs ≥ 1.50 (SeeDB, MS, BABB, THF-DBE; Fig 1A). These clearing agents rendered muscle, the joint capsule, ligament and tendon, the patella, menisci, and cortical bone (Fig 1B) largely transparent—consistent with findings in other non-musculoskeletal tissues [39,43,60]. Of the remaining clearing agents, TDE achieved decent optical clearing, rendering muscle, the joint capsule, tendon and ligament, articular cartilage, and cortical bone varying degrees of translucent. Visikol and ClearT2 had minimal to no effects on joint clarity. We note that except for BABB and THF-DBE, we were unable to achieve effective clearing of bone marrow (possibly due to the high scatter of hemoglobin in the red blood cells of marrow [78,79]), nor were we able to completely clear the thin calcified cartilage zones immediately underlying the articular cartilage and growth plates. However, based upon stereomicroscopic inspection we identified four clearing protocols (SeeDB, MS, BABB, and THF-DBE) that could appreciably clear the majority of the murine tibiofemoral joint.

The use of pair-wise, quantitative UV-Vis spectroscopy measurements on cleansed murine cortical bone samples demonstrated that all clearing agents tested, could to some extent, increase light transmission through cortical bone (Fig 2 and Table 4). However, only clearing agents with RI ≥ 1.47 (TDE, SeeDB, MS, BABB, THF-DBE) demonstrating significantly increased light transmission compared to uncleared bones (Fig 2). Similar findings were observed for the number of scattering events predicted within the bone cortex following clearing at four common visible light imaging wavelengths (405-, 488-, 562-, 633-nm; Table 5). We also observed that the decrease in tissue scattering events was inversely-correlated with clearing agent RI (Fig 3B). It should be noted that due to the cylindrical morphology of our cortical bone segments the prediction of the number of light scattering events observed in the present transmitted light spectroscopy study are approximate. However, our estimated scattering coefficient values for murine bone were largely consistent with values reported previously for bone [80]. However, since these measurements were performed in a pair-wise manner, across samples with small size and shape variances (S1 Fig), the observed relationships between optical clearing agents are internally consistent. Somewhat surprisingly, the clearing ability of the aqueous agent TDE exceeded expectations, demonstrating a clearing potential that belied its relatively low RI value (RI = 1.47; note the relatively low number of predicted scattering events in Fig 3B). One explanation for this finding may be the preferential extraction of water from bone tissue by 97% TDE (3% water) when compared to the other lower-RI aqueous clearing agents (typically 20–75% water), since the extent of water loss in collagenous tissues correlates with clearing potential [81].

Two additional confocal investigations, involving the imaging of i) dynamically labeled bone formation and ii) osteocyte lacunar-canalicular cytoarchitecture following en bloc staining, further demonstrated the benefits of optical clearing for enhancing the visualization of intact musculoskeletal tissues in situ. Optical clearing of dynamically labeled bone permitted macro-scale 3-D visualization of singly and doubly labeled bone surfaces, and thus bone formation fronts on the periosteal, endosteal, and trabecular surfaces, as well as individual bone formation “nodules”, throughout intact murine femoral bone (Fig 4 and S2 Fig). BABB, a non-aqueous clearing agent, was the most effective in allowing full thickness (from the periosteal to endosteal surfaces) 3-D imaging of dynamic labels in the femoral diaphysis using 1- and 2-photon microscopy. Dynamic labeling image quality decreased progressively in MS, SeeDB, and Visikol; however, each of these agents resulted in improvements over plastic embedded or uncleared, water immersed specimens (not shown). While this study did not quantify mineral apposition or bone formation rates, we are confident, based upon experience with murine histomorphemetry, that the qualitative results presented here are suitable for 3-D tomographic quantification of bone formation in murine bone. Given these findings we suggest that the use of optical clearing techniques for the direct visualization of bone formation in intact bone specimens can provide numerous benefits over traditional “gold-standard” histomorphmetry techniques; these being i) a reduction in preparation time from weeks to days (Table 2), ii) a reduction in the cost of materials used (Table 2), iii) an unmatched technical simplicity, and most importantly iv) amelioration of sample loss during preparation and v) the ability for contiguous tissue-level 3-D imaging. However, several limitations and considerations with the current clearing and visualization technique are acknowledged. First, delineating the periosteal and endosteal envelopes in bone regions lacking dynamic labels could be difficult, thus making the establishment of referents (areas and perimeters) problematic [82]. In this regard the use of bone autofluoresence [83] may overcome this difficulty, as well as provide information regarding the structure of the underlying bone. Second, the presence of “uncleared”, scattering and absorbing bone marrow may present challenges to imaging dynamic labels in trabecular bone, especially in larger specimens; techniques to flush the marrow from the specimens and the use of BABB and THF-DBE (which appear to better clear the marrow) may be of benefit. Third, long term processing and storage of dynamically labeled bone in aqueous clearing agents should be cautioned against. While dissolution of bone mineral, and label loss did not appear to be a significant consideration during our relatively short clearing and imaging procedures, the long-term consequences of storage in aqueous agents are unknown (see below). Non-aqueous agents (BABB and MS), which also exhibited the highest imaging quality, are not expected to cause bone mineral dissolution over time and would be a primary choice for in situ dynamic label imaging.

Optical clearing also improved the ability to visualize micro-scale osteocyte LCS detail in en bloc stained bone specimens (Figs 5 & 6). High-quality “gold-standard” 1-photon confocal imaging of the bone LCS in plastic embedded, sectioned, and polished BF stained sections was largely limited to a maximum of ~45-um in depth; a depth consistent with findings in previous 1-photon confocal studies of en bloc stained plastic embedded murine bone [17,19]. Two-photon imaging improved this imaging depth slightly to ~65-um. Use of non-aqueous optical clearing agents (MS and BABB) enhanced the depth at which BF stained LCS canalicular architecture could be visualized via 1- and 2-photon confocal microscopy 2 to 3-fold (Figs 5 & 6). LCS imaging was further improved by changing the en bloc staining technique from BF to Villaneuva’s Osteochrome. Following Osteochrome staining MS, BABB, and THF-DBE clearing agents were found to be compatible with both 1- and 2-photon confocal microscopy, while TDE and SeeDB was only compatible with 1-photon imaging (Fig 6). ClearT2 and FocusClear were found to be incompatible with Osteochrome staining and clearing (see below). Overall, the use of Osteochrome staining, BABB clearing, and 2-photon imaging resulted in upwards of a 4-fold increase in the imaging depth (>200-um) at which fine LCS canalicular details could be visualized (Figs 5 & 6), as well as allowing canalicular visualization through the full thickness of both intact murine calvarial and femoral cortices (up to 250um; S7 & S8 Movies). It is also noted that the use of optical clearing agents, specifically MS and BABB, had a significant effect on the imaging of bone lacuna at depth, permitting lacunar imaging at depths approaching 1-mm under 2-photon microscopy, as opposed to ~50 to 150-um in plastic embedded specimens (not shown). Together, these results suggest that optical clearing and imaging of en bloc stained bone specimens (specifically Osteochrome stained specimens) provided benefits over traditional “gold-standard” techniques similar to those seen in our dynamic labeling study, as well as significant improvement in LCS imaging quality and depth of penetration.

One concern regarding the use of optical clearing techniques on biological tissues is their effect on tissue and cellular structure. Indeed, several of the clearing methods used in this study have been documented to alter soft-tissue volume during clearing [38]; thus clearing agents could potentially alter the ultrastructure/architecture of musculoskeletal tissues and cells. In the present study we assessed both gross (macroscopic) changes in joint and bone structure, as well as microscopic changes in bone LCS structure following optical clearing. Non-aqueous clearing agents have previously been shown to cause soft-tissue shrinkage [40,42,84], and the use of “gentle” dehydration procedures have been suggested as a means to limit tissue deformation during processing. Our non-aqueous dehydration procedures, while relatively quick, were performed in an isometric manner to try to limit tissue structure changes. Nevertheless, in our hands we observed that BABB caused distinct muscle shrinkage, but no appreciable changes in the soft tissues of the joint proper. Neither MS or THF-DBE appeared to grossly influence the morphology of musculoskeletal tissues. Some aqueous clearing agents have been shown to cause tissue swelling; in this study only the aqueous clearing agent Visikol was found to cause gross swelling of muscle in our specimens, but no other gross changes were observed. While the effect of FocusClear on the clearing of intact joints was not assessed in this study, the literature suggests that it and its mountant, MountClear, may lead to swelling of soft tissues as well [32]. Furthermore, pair-wise qualitative measurements of femoral bone geometry prior to and following optical clearing demonstrated that none of the optical clearing techniques significantly altered the geometry of cleared bone segments (S1 Fig). This was not unexpected since the composite nature of bone is well designed to resist both the compressive and tensile forces that would required to deform bone. It is unlikely that chemical clearing could generate stresses necessary to cause gross measurable changes in bone size. Lastly, the effect of optical clearing agents on the micro-scale structure of the bone LCS was assessed by quantifying lacunar volumes and volume distributions from high-resolution confocal z-stacks of Osteochrome stained bone segments. Similar to the effects of clearing agents on gross bone geometry, our optical clearing agents were not found to have a significant effect on lacunar volume (Fig 7).

While this study demonstrated the great utility of several relatively rapid clearing techniques for improving the imaging of musculoskeletal tissue across multiple imaging and analysis modalities, the coverage of clearing agents herein, and the limits to their application is by no means comprehensive. However, based upon the present results, general recommendations for the use of optical clearing techniques in the study of murine musculoskeletal tissue can be provided. For the panel of optical clearing agents tested (RI = 1.45 to 1.56), those agents with higher refractive indexes demonstrated significantly better performance across each imaging application tested. As a result, we rate the higher-RI, non-aqueous clearing agents as overall superior over the lower-RI, aqueous clearing agents for the presented imaging applications. Additionally, these techniques were found to be quite rapid, with maximal clearance being achieved within the time guidelines presented (typically 4 to 7-days). Revisiting the samples following several weeks (to months) of maintained immersion in their respected clearing agents demonstrated no additional enhancement in clearing over the initial findings (not shown).

Despite the success of various clearing procedures in the present study, the specific clearing agents tested herein are not without their limitations. While immersion in THF-DBE resulted in the greatest clearing of musculoskeletal tissue, THF-DBE is known to quench protein fluorescence [45]. Indeed, in the present study THF-DBE appeared to quench the relatively strong fluorescent signal of Osteochrome, requiring approximately 2-fold greater laser excitation energies to obtain fluorescent emission comparable to the other methods. BABB, another well performing non-aqueous agent in the present study also has been reported to quench biological fluorescence [40,45]; however, we did not observe any adverse effects on the fluorescent imaging of chemical dyes or fluorescent bone labels. It should also be noted that chemical constituents of both THF-DBE and BABB have been described as toxic, and as peroxide formers; thus, extra care is required in their handling and long-term storage. MS, on the other hand, which demonstrated clearing capabilities comparable to THF-DBE and BABB, is minimally toxic (unless ingested), non-reactive, and suggested to be more compatible with protein fluorescence. Given the minimal differences in the processing times and optical performance among these non-aqueous clearing agents, we believe that MS (a.k.a. Murray’s clear) represents an ideal, non-aqueous, high-RI agent for clearing musculoskeletal tissues for optical imaging.

Among the aqueous clearing agents, we can only recommend the use of SeeDB and TDE for general clearing use in musculoskeletal tissues. Even over the relatively short clearing times utilized we found that ClearT2 and FocusClear cleared bones were incompatible with fluorescent imaging of chemically dyed bones. For FocusClear, this may be due to the presence of the calcium chelating agent, Ethylenediaminetetraacetic acid (EDTA), within its proprietary chemical formulation [85], which may aide in the dissolution of bone mineral and surface bound dyes. The mechanism for ClearT2’s poor imaging performance is not known; however, it is noted that one of ClearT2’s chemical constituents, formamide, is toxic and a teratogen, thus increased caution with recommended with its use. Visikol, a proprietary aqueous clearing agent, was compatible with both the use of fluorescent chemical dyes and bone labels. However, while Visikol could reasonably clear muscle and connective-tissue of murine joint in our hands, it had a marginal effect on the imaging performance in bone. Conversely, TDE and SeeDB were shown to improve the optical imaging of musculoskeletal tissues to level comparable to that of plastic-embedded samples. SeeDB has been previously shown to improve musculoskeletal imaging in bovine and murine tissues [55,56], and we observed similar improvements in 1-photon confocal imaging in the present study (up to ~50um of LCS canalicular imaging depth). While SeeBD does not affect tissue morphology and is compatible with studies involving biological fluorescence, we found it to be a less-than-ideal clearing agent for musculoskeletal tissues due its viscous nature (the viscosity of saturated fructose necessitates extended processing times and makes specimen manipulation difficult) and the fact that we found it incompatible with multiphoton microscopy techniques (SeeDB generated highly fluorescent Maillard reaction products and tissue damage under high-energy near-infrared 2-photon illumination [not shown]). On the other hand, TDE was found to be the best water miscible (aqueous) clearing agent for most of the imaging modalities tested in this study. The refractive index of TDE is tunable, it is easy to work with, does not cause soft-tissue tissue deformation, does not interfere with biological fluorescence, is relatively inexpensive, and is non-toxic [38,41]. However, we must note that despite decent 1-photon imaging of Osteochrome-stained, TDE-cleared bone samples (comparable to that of “gold-standard” 1-P plastic-embedded imaging), TDE appeared to hamper LCS imaging under 2-photon microscopy.

Lastly, we remind researchers that the recommendations provided herein for the optical clearing of murine musculoskeletal tissues should be taken as guidelines for the selection and use of clearing agents in musculoskeletal tissues. The final selection of clearing agents will be determined by the needs and constraints of a researcher’s specific experiments and should be validated prior to full-scale implementation. While all of the recommended clearing protocols are of relatively short duration (~2-14-days depending on clearing procedure and specimens size), processing times may require optimization if tissues from larger animal models are utilized. Additionally, the limits on imaging depth presented in this study should also be taken as general guidelines since deep-tissue imaging quality is highly dependent on microscope setup and choice of objectives. While a long working-distance (1.7mm), high numerical aperture (1.0) objective was utilized in this study, optical correction for the specific mounting agents utilized was not available, thus, the influence of optical aberrations likely reduced our overall imaging quality. Additionally, scan settings were established to achieve a balance between 3D scan speed (on the order tens of minutes) and image quality. Presumably, better image quality and imaging penetration could be achieved with specially designed objectives [32], longer scan durations, and the advent of increasingly sensitive confocal/multiphoton microscopy devices.

In conclusion, this study demonstrated that simple, straightforward, and inexpensive modifications to tried-and-true en bloc imaging preparation procedures, namely optical clearing, could facilitate dramatic improvements in the 3-D imaging of intact musculoskeletal tissues and cells in situ. Whereas we expected specific clearing protocols to be effective in rendering isolated bone samples optically clear and thereby enhance in situ imaging, we were enthusiastically surprised by the ability of several of these techniques to render complex, multi-component, multi-RI musculoskeletal tissues (murine knee joints) optically clear as well. Presently, the use of clearing agents in musculoskeletal tissue was limited to 1- and 2-photon confocal microscopy techniques; thus, the largest hurdle to their use in the high-throughput 3-D visualization and quantification of musculoskeletal architecture is the time required to capture large, high-resolution, 3-D images of intact tissues. However, recent work combining optical clearing with advanced imaging technologies, such as light sheet microscopy, have demonstrated the acceleration of data collection time by orders of magnitude [45,86,87] and may help to overcome these hurdles. While the concept of optical clearing is not new to the field of musculoskeletal biology (glycerin-based clearing and mounting of alcian blue and alizarin red stained skeletons has been utilized in studies of skeletal development studies for decades [88–90]), it is only recently that modern clearing methods have been directly applied to the high-resolution study of adult musculoskeletal tissues [55–57]. The rapid acceptance and success of these techniques within the fields of neuroscience and developmental biology suggest that the combination of optical clearing and advanced imaging, can complement, if not supplant, classical musculoskeletal analysis techniques. Specific areas where optical clearing and advanced bioimaging techniques may prove extremely beneficial to the musculoskeletal field include, but are not limited to, i) tomographic analysis of bone growth [30,91], ii) in situ bone microdamage assessment [92], iii) in situ soft-tissue characterization and damage imaging [93–95], and iv) fluorescence based in situ lineage tracing [96,97], cell tracking [98–100], and protein/macromolecular labeling and localization [21,101]. Given their past successes across multiple research fields, we believe that optical clearing techniques have the potential to transform the in situ investigation and quantification of musculoskeletal physiology and pathophysiology.

## Supporting Information

S1 Fig Quantification of effect of optical clearing on bone tissue morphology. The anterior-posterior, and medial-lateral width of non-decalcified femoral bone segments were measured, using digital calipers, prior to and following optical clearing. Immersion in optical clearing agents, till maximally cleared, had no effect on the measured bone morphology (paired t-test, GraphPad Prism, p<0.05; n = 3–4 segments/clearing agent).
(TIF)

S2 Fig 2. 3-D representation of dynamically labeled bone from various regions within a femur from an adolescent mouse that had been administered calcien green (green) and alizarin red complexone (red) 13- and 3-days prior to sacrifice, respectively. 3-D image stacks of dynamically labeled bone were acquired for anterior-lateral regions of the mouse femur (shown in the red and blue boxes). Dynamically labeled appositional fronts were clearly visualized on both the periosteal (P) and endosteal (E) surfaces of compact cortical bone in the mid diaphysis (left side of panel). In addition, labeled surfaces were clearly present on compact and trabecular bone within more complex regions of the murine femur, such as the third trochanter—the region where the ascending *Musculus glutaeus superficialis* attaches to the femur (Favier 1996 Development; Hamrick 2000 Bone) (right side of panel). Reconstructions of six separate z-stack scans are shown.
(TIF)

S1 Methods Supplemental materials and methods. (DOCX)

S1 Movie Dynamic labeling on the endosteal surface of anterior-medial femoral mid-diaphysis. (MOV)

S2 Movie Dynamic labeling through the full thickness of the anterior femoral mid-diaphysis. (MOV)

S3 Movie Dynamic labeling on the periosteal surface of anterior-lateral femoral mid-diaphysis. (MOV)

S4 Movie Dynamic labeling of the bone formation in the central portion of the third trochanter of the femur. (MOV)

S5 Movie Dynamic labeling of the bone formation in the distal portion of the third trochanter of the femur. (MOV)

S6 Movie Dynamic labeling of the bone formation along the bone ridge distal to the third trochanter of the femur. (MOV)

S7 Movie Confocal z-stack demonstrating the ability to perform full, through-thickness imaging of bone LCS cytoarchitecture and morphology in an Osteochrome stained murine anterior femoral mid diaphysis (40x, image width = 212um). (MOV)

S8 Movie Confocal z-stack demonstrating the ability to perform full, through-thickness imaging of bone LCS cytoarchitecture and morphology in an Osteochrome stained murine calvaria (40x, image width = 212um). (MOV)
