# Supplementary figures and images for: Seeing through Musculoskeletal Tissues: Improving In Situ Imaging of Bone and the Lacunar Canalicular System through Optical Clearing
Source: PLoS One. 2016 Mar 1;11(3):e0150268. doi: 10.1371/journal.pone.0150268 (PMC4773178; doi:10.1371/journal.pone.0150268)

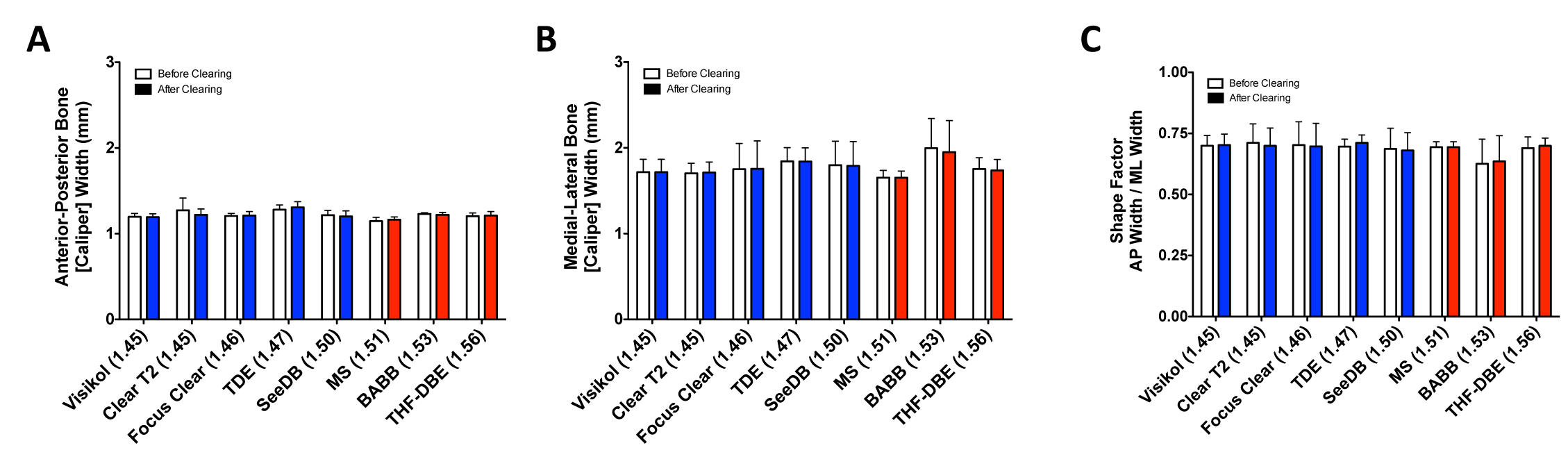

Supplement: S1 Fig — The anterior-posterior, and medial-lateral width of non-decalcified femoral bone segments were measured, using digital calipers, prior to and following optical clearing. Immersion in optical clearing agents, till maximally cleared, had no effect on the measured bone morphology (paired t-test, GraphPad Prism, p<0.05; n = 3–4 segments/clearing agent). (TIF) [file pone.0150268.s001.tif]

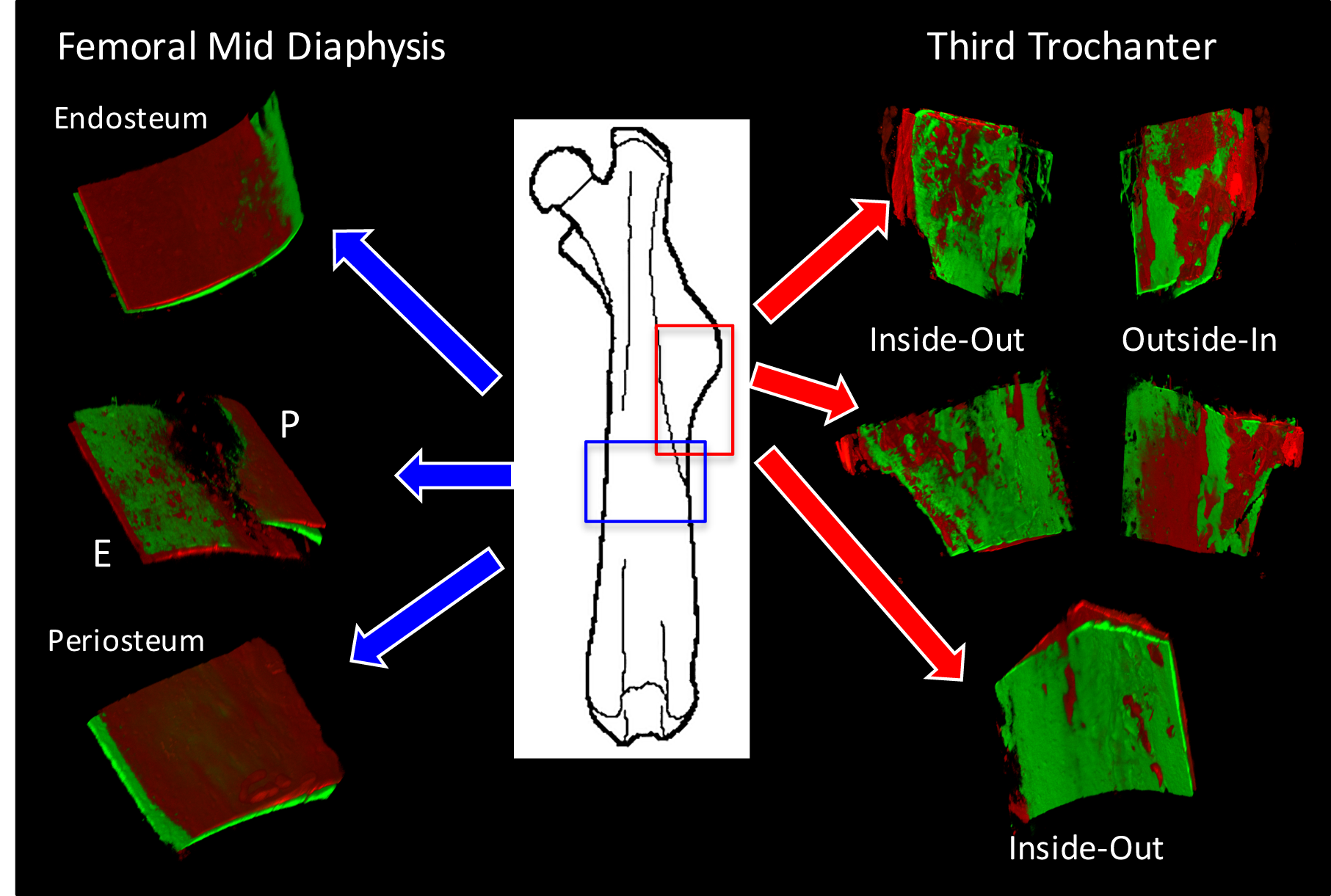

Supplement: S2 Fig — 3-D image stacks of dynamically labeled bone were acquired for anterior-lateral regions of the mouse femur (shown in the red and blue boxes). Dynamically labeled appositional fronts were clearly visualized on both the periosteal (P) and endosteal (E) surfaces of compact cortical bone in the mid diaphysis (left side of panel). In addition, labeled surfaces were clearly present on compact and trabecular bone within more complex regions of the murine femur, such as the third trochanter—the region where the ascending Musculus glutaeus superficialis attaches to the femur (Favier 1996 Development; Hamrick 2000 Bone) (right side of panel). Reconstructions of six separate z-stack scans are shown. (TIF) [file pone.0150268.s002.tif]
