# Supplementary material for: Seeing through Musculoskeletal Tissues: Improving In Situ Imaging of Bone and the Lacunar Canalicular System through Optical Clearing
Source: PLoS One. 2016 Mar 1;11(3):e0150268. doi: 10.1371/journal.pone.0150268 (PMC4773178; doi:10.1371/journal.pone.0150268)
Supplement: S1 Methods — (DOCX) [file pone.0150268.s003.docx]

**S1 Methods: Supplemental materials and methods.**

**En bloc Staining**

Basic fuschin (BF) – Following fixation, musculoskeletal specimens in the BF arm of the study were simultaneously stained and dehydrated, non-decalcified in increasing concentrations of ethanol containing BF (26) BF stock solution consisted of 1% basic fuchsin in 100% ethanol (wt./vol.; hereby referred to 100% BF). Samples were stained at room temperature according to the following schedule: 80% BF stock solution in water (80% BF + 20% water, vol./vol., 24-hours), 80% BF (24-hr), 90% BF (24-hr), 90% BF (24-hr), 100% BF (24-hr), 100% BF (24-hr), respectively. Following a wash in 100% ethanol (30-min) samples were then directed down selected non-aqueous clearing pathways.

Osteochrome - Specimens in the osteochrome arm of the study were, according to the manufactures directions, stained in Villaneuva’s Osteochrome Bone Stain (Polysciences, Inc., Warrington, PA) for 48-hr at room temperature following fixation, they were then washed in 1% detergent in water (vol./vol.) for 30-min, differentiated in 0.01% acetic acid in 95% methanol (30-min) before being directed down select aqueous and non-aqueous clearing pathways.

Dynamic bone labels – Tissues in the dynamic bone label arm of the study were left unstained following fixation and were directed down select non-aqueous and aqueous clearing pathways.

**Optical Clearing**

Note: for the following clearing steps all times, in parentheses, are given as minimal times for the murine bone segments utilized within our study, longer times (~3-4x longer) were used for the intact joints, and optimization of processing times may be required for larger specimens. For each step processing/clearing was initiated following fixing, washing, and staining (as required) and was performed in an excess of reagent, typically 1-4mL depending on specimen size. All steps were performed at room-temperature, unless otherwise noted.

**Aqueous clearing**

**Visiko**l - Samples in this arm of the study were directly transferred to an excess volume of the proprietary clearing agent Visikol (Photosys, LLC., New Brunswick, NJ). Visikol is one-step clearing agent, thus specimens remained immersed in Visikol until they achieved maximal clearance (~12+-hrs). Samples were then mounted in fresh Visikol prior to imaging.

**ClearT2** – Clearing in ClearT2 (35) involved the sequential immersion of samples in solutions of 25% formamide + 10% polyethylene glycol (PEG) vol./vol. in PBS (4-hrs), 50% formamide + 20% PEG in PBS (ClearT2; 4-hrs), fresh ClearT2 (24-hours or until cleared), and were then mounted in fresh ClearT2.

**FocusClear** – FocusClear (Cedarlane, Burlington, NC) is another proprietary, one-step clearing agent. Following the manufactures directions, clearing in FocusClear was performed by immersing the samples in FocusClear at room temperature until clear (at least 48-hours) followed by mounting in the proprietary mounting agent, MountClear, for imaging.

**SeeDB** – Clearing in SeeDB (36) involved immersion of specimens in increasing concentrations of fructose in water. Samples were immersed in 20% fructose wt./vol. (8-hrs), 40% fructose (8-hrs), 60% fructose (8-hrs), 80% fructose (8-hrs), 100% fructose (8-hrs) at 4° Celsius, followed by immersion and mounting in 80.2% wt/wt fructose in water (SeeDB) at 50° Celsius prior to imaging (~8hrs). All fructose solutions contained α-thioglycerol (0.5% vol./vol.) to “prevent browning and autofluorescence accumulation as a result of the Maillard reaction” that can occur during heating (36) as well as sodium azide (0.01% wt./vol.) as a preservative and biocide.

**TDE** – Clearing in TDE (34,37) involved the sequential immersion of the samples in increasing concentrations of 2,2’-thiodiethanol in water. Samples were immersed in 10% TDE vol./vol. (12-hrs), 25% TDE (12-hrs), 50% TDE (12-hrs), and 97% TDE (12-hrs). The concentration of TDE in water can be varied to change the final solutions refractive index; 97% TDE corresponds to a RI of 1.47 (34). Thus, specimens were immersed in 97% TDE until clear (~12-hrs), and then mounted in 97% TDE for imaging.

**Non-aqueous clearing**

**MS** – Following dehydration in graded ethanols; 70% EtOH vol./vol. (4-hr), 80% EtOH (4-hr), 80% EtOH (4-hr), 90% EtOH (4-hr), 95% EtOH (4-hr), 100% EtOH (4-hr), 100% EtOH (4-hr), the samples were immersed sequentially in 2-propanol (2 changes, 2-hrs each), methyl salicylate (MS: aka Murray’s Clear) (2 changes, 4-hrs each till clear), and then mounted in fresh MS prior to imaging (39).

**BABB** – Following dehydration in graded ethanols (as immediately above), samples were immersed in hexane (12-hrs), and then 2 changes of benzyl alcohol-benzyl benzoate (BABB; 1:3 vol./vol.) for 12-hrs each or until the samples were clear and sank, followed by mounting in fresh BABB (40,41).

**THF-DBE** – During THF-DBE processing samples were dehydrated in increasing concentrations of tetrahydrofuran (THF; a cyclic ether solvent) in water at room temperature; 50% THF vol./vol. (12-hrs), 70% THF (12-hrs), 80% THF (12-hrs), 100% THF (12-hrs), 100% THF (12-hrs), followed by immersion in dibenzyl ether (DBE) until clear (24-hrs, or till clear). Samples were then mounted in fresh DBE (38) prior to imaging.

**Plastic embedding**

A subset of samples was imbedded in methyl methacrylate (acrylic) following non-aqueous processing (26). Following dehydration in graded ethanols (as above), samples were immersed sequentially in 2-propanol (2 changes, 2-hrs each) and methyl salicylate (MS) (2 changes, 4-hrs each till clear), and then infiltrated in three changes (24-hrs each) of methyl methacrylate containing the plasticizing agent n-butyl phthalate (15% vol./vol.) and increasing concentrations of the polymerization catalyst dry benzoyl peroxide (0-2.0% wt./vol.) at 4° Celsius. Following infiltration specimens were placed into glass scintillation vials containing thickened methyl methacrylate and solid methyl methacrylate base, and polymerized in a water bath at 40° Celsius. Following polymerization, the samples were liberated from the scintillation vials and prepped, via block facing, thick sectioning using a low speed saw, and surface polishing, for en bloc imaging.

**Imaging chambers**

Custom imaging chambers were constructed for stereomicroscopy as well as upright confocal microscopy. The stereomicroscopy chamber consisted of a specimen holder and glass-bottomed open-top bath, made of non-reactive machined aluminum, that allowed the intact joints to be positioned for both anterior and lateral viewing while remaining immersed within the respective clearing agents. For confocal microscopy different sized custom imaging wells were constructed out of the non-reactive material polydimethylsiloxane (PDMS; SYLGARD 184, Dow Corning). For upright microscopy the PDMS wells were placed atop a 3-inch by 3-inch glass slide in order to allow complete immersion of the bone specimens within wells containing their respective clearing and mounting agents. The wells were then covered with a #1.5 glass coverslip to create a temporary imaging chamber for upright confocal imaging. For inverted microscopy PDMS wells were placed atop the coverglass base of Lab-Tek chambered coverglass slides, which allowed the bone specimens to be completely immersed in their respective clearing and mounting agents while being imaged from below.

**Confocal Microscopy Imaging**

Three-dimensional (3-D) deep-tissue imaging was performed through the capture of z-stacks using laser scanning confocal microscope (LSCM). Deep-tissue images were obtained using a high, 1.0-numerical aperture water immersion lens (W Plan-Apochromat 20x) with a 1.7mm working distance and corrected for a #1.5 coverslip (0.17mm thickness) on a upright Zeiss LSM780NLO LSCM with spectral detection and capable of 1- and 2-photon imaging. Higher resolution imaging was also performed on an inverted Zeiss LSM880 LSCM with 1-photon spectral detection using a high, 1.3-numerical aperture oil lens (Plan-Apochromat 40x). Images were typically captured at 1024x1024-pixels under 1.0x to 6x zoom (0.414- to 0.069-um/pixel, respectively. Images were captured with a relatively fast, 0.39 to 0.79-us/pixel dwell-time and line averaging (4-8 fold) was implemented to improve the signal-to-noise ratio and image quality. Detector gain typically was set between 700-800 units and the detector offset was adjusted to maximize the dynamic range within the images. Excitation parameters for either 1- or 2-photon illumination, as well as for emission capture were established individually for each stain/fluorophore and illumination modality. For one-photon (1-P) microscopy the following excitation/emission parameter sets were used: calcein green-alizarin red complexone dynamic labels – Ex 488/561-nm (typically 0.5-2.0% laser power) & Em 490-560/604-656nm; basic fuschin – Excitation (Ex) 561-nm (0.8-1.8% laser power) & Emission (Em) 560-690-nm; osteochrome – Ex 561-nm (0.5-4.0% laser power) & Em 560-690-nm. For two-photon (2-P) microscopy a Ti-sapphire multiphoton light source (Chameleon Vision II, Coherent Inc., Santa Clara, CA) and the LSM780 NLO module was used to collect images under the following excitation/emission conditions: calcein green-alizarin red complexone dynamic labels – Ex 750-nm (10% laser power) & Em 490-569/587-656nm*;* basic fuschin – Ex 740-nm (3.0-7.5% laser power) & Em 578-665-nm; osteochrome – Ex 740-nm (1.5-6% laser power) & Em 587-674nm. During both 1- and 2-P imaging laser intensity compensation (within the powers indicated) was used to maintain consistent illumination as the depth of the imaging plane increased, while also attempting to minimize photobleaching. Optical slice thickness was set to between 0.5-0.8 um for the collection of all slices in the 3-D stacks.
